# An Integrated Strategy for Effective-Component Discovery of Astragali Radix in the Treatment of Lung Cancer

**DOI:** 10.3389/fphar.2020.580978

**Published:** 2021-01-14

**Authors:** Bing Yang, Nan Yang, Yaping Chen, Maomao Zhu, Yuanpei Lian, Zhiwei Xiong, Bei Wang, Liang Feng, Xiaobin Jia

**Affiliations:** ^1^ School of Traditional Chinese Pharmacy, China Pharmaceutical University, Nanjing, China; ^2^ Nanjing University of Chinese Medicine, Nanjing, China; ^3^ State Key Laboratory of Natural Medicines, China Pharmaceutical University, Nanjing, China

**Keywords:** key word: effective components, traditional Chinese medicine, astragali radix, lung cancer, autophagy, potential targets, network pharmacology, cell membrane immobilized chromatography

## Abstract

Lung cancer is one of the most devastating diseases worldwide, with high incidence and mortality worldwide, and the anticancer potential of traditional Chinese medicine (TCM) has been gradually recognized by the scientific community. Astragali Radix (AR) is commonly used in traditional Chinese medicine in the treatment of lung cancer and has a certain clinical effect, but effective components and targets are still unclear. In the study, we established an integrated strategy for effective-component discovery of AR in the treatment of lung cancer based on a variety of techniques. First, the effective components and potential targets of AR were deciphered by the “component-target-disease” network using network pharmacology, and potential signal pathways on lung cancer were predicted by Gene Ontology (GO) biological function enrichment analysis and Kyoto Encyclopedia of Genes and Genomes (KEGG) enrichment analyses. Then, the therapeutic effects of AR in the treatment of lung cancer were evaluated *in vivo* using A/J mice, and the potential targets related to autophagy and potential signal pathway were verified by Western blot analysis, immunofluorescence staining, and real-time PCR technology at protein and gene expression level. Finally, metabolism *in vitro* by rat intestinal flora and cell membrane immobilized chromatography technology were used to screen the effective components of AR in the treatment of lung cancer, and remaining components from the cell immobilized chromatography were collected and analyzed by ultra-performance liquid chromatography–electrospray quadrupole time-of-flight mass spectrometry (UPLC-Q-TOF-MS). The screening results of the integrated strategy showed that calycosin-7-O-β-D-glucoside, ononin, calycosin, astragaloside IV, astragaloside II, cycloastragenol, and formononetin may be effective components of AR in the treatment of lung cancer, and they may play a role in the treatment of lung cancer through autophagy and p53/AMPK/mTOR signaling pathway. The integrated strategy for effective-component discovery provided a valuable reference mode for finding the pharmacodynamic material basis of complex TCM systems. In addition, the prediction for targets and signal pathways laid a foundation for further study on the mechanism of AR in the treatment of lung cancer.

## Introduction

As a global health burden, lung cancer is the highest incidence cancer at present (Torre et al., 2015; Wang et al., 2019). According to relevant statistics, lung cancer accounts for 19.4% of cancer-related deaths with 1.59 million deaths each year, which seriously endangers human health (Ke et al., 2019). As estimated by the International Agency for Research on Cancer, the number of deaths caused by lung cancer will raise to 10 million deaths per year by 2030 (Lou et al., 2016). However, the existing therapies, including radiotherapy, chemotherapy, and the emerging target therapy, are still unsatisfactory to improve the survival of lung cancer patients during the last 30 years (Cheng et al., 2018). So, it is an urgently required issue to achieve a breakthrough in medical treatment of lung cancer.

In recent years, the anticancer potential of traditional Chinese medicine (TCM) has been gradually recognized by the scientific community (Jiang et al., 2017; Zhu et al., 2019). In China, TCM is widely used in the treatment of cancer by preventing tumorigenesis, attenuating the toxicity, enhancing the therapeutic effect of radiotherapy and chemotherapy, reducing tumor recurrence, *etc*. (Wang et al., 2018; Zhang et al., 2018). Astragali Radix (AR), a well-known TCM, is the dried root of *Astragalus membranaceus* (Fisch.) Bge. var. *mongholicus* (Bge.) Hsiao or *Astragalus membranaceus* (Fisch.) Bge. has been used as a common clinical medicine in China for thousands of years. The accumulated data showed that AR was beneficial for the treatment of lung cancer in clinical practice (He et al., 2013; Xiao et al., 2019). However, the effective components and potential targets of AR in the treatment of lung cancer have not been reported.

Network pharmacology is a discipline for investigating pathogenesis of disease through constructing and analyzing biological networks (Chen et al., 2019), and provides a powerful tool for screening effective components and potential targets of TCM by establishing a “component-target-disease” network. Modern pharmacological research has proved that the combination of drugs with the lipid bilayer, receptors, and enzymes on the cell membrane is a main mechanism of drug action. Therefore, the effective components can be screened according to the affinity between the components and the cell membrane. Cell membrane immobilized chromatography, as a kind of cell membrane chromatography, uses active cells as the separation vector, TCM extracts as the object, and the separation was carried out according to whether the ingredients in the extract have specific affinity with the cells. In the study, we established an integrated strategy for effective-component discovery of AR in the treatment of lung cancer based on a variety of techniques, and investigated the relationship between autophagy and the anticancer effect of AR *in vitro* and *in vivo*.

## Materials and Methods

### Materials and Reagents

AR was obtained from Anhui Jingquan Group Herbal Pieces Co., Ltd (Anqing city, Anhui Province, China) and was identified by De-kang Wu (professor of Nanjing University of traditional Chinese medicine) as the dried root of *Astragalus membranaceus* (Fisch.) Bge. var. *mongholicus* (Bge.). Benzopyrene was purchased from Aladdin Reagent Co., Ltd. (Shanghai, China). Cisplatin (DDP, 1 mg/ml, 20 ml) was gained from Nanjing pharmaceutical factory Co., Ltd. (Nanjing, China). 4% paraformaldehyde fixative was purchased from Nanjing Nanao Technology Co., Ltd. (Nanjing, China). Hematoxylin dyeing solution (D005) was purchased from Nanjing Jiancheng Bioengineering Institute (Nanjing, China). Eosin dye solution (KGA231), 0.25% trypsin, 1% Triton X-100, goat serum, 4’,6-diamidino-2-phenylindole, and DMEM high glucose medium were purchased from Nanjing KeyGen Biotech logical Co., Ltd. (Nanjing, China). Glyceraldehyde-3-phosphate dehydrogenase (GAPDH, 0802) antibody was purchased from Shanghai Kangcheng Biological Engineering Co., Ltd. (Shanghai, China). The antibodies against protein 53 (p53, ab131442), phosphorylated B-cell lymphoma-2 (p-Bcl-2, ab138406), mammalian target of rapamycin (mTOR, ab2732), AMP-activated protein kinase (AMPK, ab32047) and Beclin1 (ab133357) were obtained from Abcam (Cambridge, United Kingdom). NP-40 lysis buffer (1210600) was purchased from Nanjing Shengxing Biotechnology Co., Ltd. (Nanjing, China). Polyvinylidene fluoride (k8JN62911) was purchased from Millipore Corporation (Bedford, MA, USA). Pre-stained protein marker was purchased from Fermentas (Burlington, Canada).

Astragaloside I (MUST-16012906) and astragaloside II (MUST-16031010) were purchased from manster biotechnology Co., Ltd. (Chengdu, China). Cycloastragenol (HHQC20170921) was purchased from Nanjing Spring and Autumn Biological Engineering Co., Ltd. (Nanjing, China). Astragaloside IV (1107781-201616), calycosin (111920-201304), calycosin-7-O-β-D-glucoside (111920-201505), formononetin (111703-200603), and ononin (111703-200501) were purchased from the National institute for Food and Drug Control of China (Beijing, China). LC-MS grade methanol and acetonitrile were purchased from Merk Company (Darmstadt, Germany). HPLC grade formic acid with a purity of 99% was purchased from Anaqua chemical supply (ACS, Houston, USA). Purified water was prepared from a Milli-Q water purification system (Millipore Corporation, Bedford, MA, USA). Other chemicals (reagent grade) used were purchased from Nanjing Chemical Reagent Co, Ltd. (Nanjing, China).

### Database Construction

All components of AR were obtained and cross-validated from TCMSP (http://lsp.nwu.edu.cn/tcmsp.php) database (Ru et al., 2014), ETCM (http://www.nrc.ac.cn:9090/ETCM/) (Xu et al., 2019), TCMID (http://www.megabionet.org/tcmid/) (Xue et al., 2012), CNKI (https://www.cnki.net/), PubMed (https://www.ncbi.nlm.nih.gov/pubmed), and SciFinder (https://scifinder.cas.org), and saved in SDF and Canonical SMILES structure format. All targets related to components of AR were collected from several databases, including PharmMapper (http://www.lilab-ecust.cn/pharmmapper/) (Wang et al., 2017), similarity ensemble approach (SEA, http://sea.bkslab.org/) (Gu and Lai, 2020), and Swiss Institute of Bioinformatics (SIB, http://www.swisstargetprediction.ch/) (Gfeller et al., 2014), and were limited to homo sapiens. All obtained targets were retrieved from GeneCards (http://www.genecards.org/) (Fishilevich et al., 2016) and Therapeutic Target Database (TTD, http://bidd.nus.edu.sg/group/cjttd) (Li et al., 2018) to explore their function to confirm if related to lung cancer, as only targets related to lung cancer can be used in subsequent studies, and named as potential targets. All components related to lung cancer were obtained by matching targets related to lung cancer with components of AR, and named as potential effective components.

### Network Construction

The potential target-effective component network was constructed using Cytoscape software (version 3.6.1). In order to further explain the mechanism of AR in the treatment of lung cancer, the protein–protein interaction (PPI) network of lung cancer–related targets was explored by STRING (version 10.5, https://string-db.org/), and was visualized with Cytoscape software (version 3.6.1). Gene Ontology (GO) and Kyoto Encyclopedia of Genes and Genomes (KEGG) enrichment analyses of potential targets were performed using the plugin ClueGo (version 2.5.4) from Cytoscape software (version 3.6.1). The signal pathways closely related to lung cancer were statistically analyzed, and those with *p* < 0.01 after Benjamin's correction were considered to be significantly changed and selected for further research, named as potential signal pathways. Then, the potential signal pathways were evaluated by occurrence frequency of potential targets.

### Preparation of Astragali Radix Sample and Component Characterization

AR sample was gained by reflux extraction twice with ten times (w/v) 70% ethanol (v/v) in thermostatic water bath for 1.5 h. The two parts of extracts were combined, and the solvent was removed by rotary evaporation to obtain dried powder of AR. The dried powder of AR was stored in 4 °C refrigerator before use. The AR sample was analyzed by ultra-performance liquid chromatography–electrospray quadrupole time-of-flight mass spectrometry (UPLC-Q-TOF-MS) to characterize chemical component, and main signals in chromatograph were identified, and compared with the reference substances. Eight components, including calycosin-7-O-β-D-glucoside, ononin, calycosin, formononetin, astragaloside IV, astragaloside II, astragaloside I, and cycloastragenol, were accurately identified. The component information and typical total ion chromatogram (AR and reference substances) were shown in Supplementary Material S1.

### Cigarettes Extract Preparation

A vacuum pump was used to simulate the human lung; a cigarette with the filter (containing 11 mg tar and 1.1 mg nicotine) was ignited and the end of the cigarette attached to a rubber hose. Smoke emitted from the burnt cigarette was dissolved in the serum-free culture medium through a glass pipette tip by a vacuum pump (Supplementary Figure S1). The smoke-solubilized serum-free culture medium was filtered by 0.22 μm sterile microporous membrane to obtain CSE.

### Animals and Experiment Design

Forty male A/J mice, weighing 18–20 g, were purchased from Shanghai Experimental Animal Research Center (License number SCKK (HU) 2013-0056, Shanghai, China) and housed under pathogen-free environment with a 12h/12 h light–dark cycle and fed with food and water *ad libitum*. All animal experiments were conducted in accordance with the guidelines of the laboratory animals and approved by the Animal Ethics Committee of Nanjing University of Chinese Medicine.

All the mice were randomly assigned to five groups: control group, model group, DDP group, 5.2 g/kg AR group, and 2.6 g/kg AR group. Except for the control group, the lung cancer model of several groups were created by intraperitoneally injecting with benzopyrene (100 mg/kg body weight dissolved in corn oil) twice a week for 4 weeks; the DDP group were intraperitoneally injected with DDP; mice in the 5.2 g/kg AR group and 2.6 g/kg AR group were orally administrated with AR at the dose of 5.2 g/kg/d and 2.6 g/kg/d (dose conversion according to the Chinese pharmacopoeia 2020 edition part one), respectively, while the control group was given normal saline solution by gavage. After that, all the groups continued to receive different treatments for 28 weeks and once a day. After 28 weeks, all mice were sacrificed, and the lungs were collected. Immediately, a portion of lung tissue was snap-froze in liquid nitrogen and stored at −70 °C for further analysis, and another portion was fixed in 4% paraformaldehyde for histopathological study.

### Histopathological Study

The lung tissues were fixed in 4% paraformaldehyde for 24 h, dehydrated and paraffin-embedded. The paraffin-embedded lung tissues were sectioned and stained with hematoxylin-eosin (HE) and immunohistochemistry (IHC). For IHC analysis, the paraffin-embedded lung tissue sections were deparaffinized in xylene and rehydrated through graded alcohol. Then, 3% H_2_O_2_ was added to block endogenous peroxidase activity. Finally, sections were blocked with normal goat serum for 30 min at room temperature and then incubated with anti-p-Bcl-2 and anti-p53 antibodies at 4 °C overnight. Sections were counterstained with hematoxylin and observed under light microscopy (Olympus, Tokyo, Japan) using ×400 magnification.

### NHBE Cell Culture and Treatments

Primary normal human bronchial epithelial (NHBE) cells were purchased from Beina Chuanglian Biotechnology Co., Ltd. (Beijing, China). NHBE cells were seeded into a plastic culture flask containing DMEM, and then placed in a humidified incubator containing 5% CO_2_ at 37 °C. Culture medium was changed every two days, until NHBE cells reached 90% confluence. NHBE cells were digested by 0.25% trypsin and 0.02% EDTA solution, and prepared for the experiment with a density of 1×10^6^ cells/mL. An MTT assay was used to assess the viability of NHBE cells exposed to different concentrations of CSE and AR (Supplementary Figure S2). According to the screening results, 10% CSE was chosen as the modeling concentration, and 1000 μg/ml and 500 μg/ml were chosen as the AR administration concentration. NHBE cells were divided into five groups (six wells per group): control groups (no treatment), model group (10% CSE), DDP groups (10% CSE+10 µg/ml DDP), 1000 µg/ml AR group (10% CSE + 1000 µg/ml AR), and 500 µg/ml AR group (10% CSE + 500 µg/ml AR). All cells were cultured in an incubator at 37 °C with 5% CO_2_ for 24 h.

### Transmission Electron Microscope Observation

NHBE cells were digested with 0.25% trypsin to prepare single-cell suspension, centrifuged at 1000 rpm for 10 min, washed twice with prechilled PBS, and then fixed with prechilled 2.5% glutaric acid for 90 min at 4 °C. After washing in PBS, the cells were fixed with 1% osmium tetroxide at 4 °C for 30 min. After fixing, the cells were dehydrated with gradient ethanol and acetone, and then embedded in Epon812 resin (Sigma). The embedded blocks were cut into ultrathin sections using an ultramicrotome, and stained with uranyl acetate and lead citrate for ultrastructural examination under transmission electron microscopy (JEOL, Tokyo, Japan).

### Western Blot Analysis

The NHBE cells were collected and used for Western blot analysis. The protein was extracted on ice using NP-40 lysis buffer (1% NP-40, 150 mM NaCl, 50 mM Tris-HCl, pH 8.0). Lysates were centrifuged at 13,000 rpm for 10 min at 4 °C, and the supernatants were collected. Protein concentration was measured by using the DC protein assay (Bio-Rad). The separated protein samples were separated by SDS-polyacrylamide gel electrophoresis and then transferred onto polyvinylidene fluoride membrane (Millipore Corporation, Billerica, MA, USA). Membranes was blocked with 5% nonfat dry milk in Tris-buffered saline with 0.05% Tween-20 (TBST) buffer, and then incubated with primary antibodies against p53 (1:500), p-Bcl-2 (diluted: 1:1000), Beclin 1 (diluted: 1:1000), AMPK (diluted: 1:1000), mTOR (diluted: 1:1000), and GAPDH (1:10,000) overnight at 4 °C. Subsequently, the membranes were incubated for 2 h at room temperature with secondary antibodies (1:10,000). Finally, the antigen–antibody complexes were visualized using enhanced chemiluminescence (Amersham) according to the manufacturer’s instructions, and visualized using Azure c600 imaging system (Azure Biosystems, Dublin, CA, USA). The protein levels were expressed as relative integrated intensity and were normalized to that of GAPDH.

### Immunofluorescence Staining

The NHBE cells were washed three times with cold PBS and fixed by 4% paraformaldehyde for 15 min at room temperature, followed by permeabilization process with 1% Triton X-100 (Fisher). NHBE cells were subsequently immuno-stained with primary antibody for 2 h at room temperature and followed by fluorescein isothiocyanate-labeled secondary antibodies for 30 min at room temperature. The samples were washed twice, adhered onto coverslips, and mounted with 4’,6-diamidino-2-phenylindole–containing mounting medium. Acquisition of images was performed using a fluorescence microscope (Leica Microsystems, Wetzlar, Germany).

### Real-Time PCR

Total RNA was extracted using the Trizol reagent (Invitrogen) according to the manufacturer's instructions. The RNA concentrations were determined using a spectrophotometer (ThermoFisher, Waltham, MA, USA). cDNA was synthesized from RNA (2 μg) using First-strand cDNA Synthesis Kit (Thermo Fisher Scientific, Waltham, MA, USA). Real-time PCR analyses were conducted to quantitate p-53, p-Bcl-2, Beclin1, AMPK, and mTOR relative expression using SYBR Green real-time PCR kit (TaKaRa, Dalian, China) with GAPDH as an internal control. The cycle threshold values from all quantitative real-time PCR experiments were analyzed using 2-^ΔΔCT^ method, and were automatically determined by the ABI 7500 Real-Time PCR System (Applied Biosystems, USA). The primers used for real-time PCR analysis were as follow: p-53, forward primer 5′-CAG​ACA​GGC​TTT​GCA​GAA​TG-3′, reverse primer 5′-GAC​CCT​GGC​ACC​TAC​AAT​GA-3’; p-Bcl-2, forward primer 5′-AAG​CTG​TCA​CAG​AGG​GGC​TA-3′, reverse primer: 5′-CAGGCTGGA AGGAGAAGATG-3’; Beclin1, forward primer 5′-GTC​CAC​GCT​CGA​CCT​TCT​TAC-3′, reverse primer 5′-CAC​TTG​CCA​GTC​TTA​ACC​TCT​G-3’; AMPK, forward primer 5′-TGC​GTG​TAC​GAA​GGA​AGA​ATC​C-3′, reverse primer 5′-TGT​GAC​TTC​CAG​GTC​TTG​GAG​TT-3′; mTOR, forward primer 5′-CAG​TTC​GCC​AGT​GGA​CTG​AAG-3′, reverse primer 5′-GCT​GGT​CAT​AGA​AGC​GAG​AC-3′; and GAPDH, forward primer 5′-AGG​TCG​GTG​TGA​ACG​GAT​TTG-3′, reverse primer 5′-TGT​AGA​CCA​TGT​AGT​TGA​GGT​CA-3′.

### Metabolism *in vitro* of Rat Intestinal Flora

#### Preparation of Anerobic Culture Medium

CaCl_2_ (0.2 g) and MgSO4.7H_2_O (0.2 g) were dissolved on 800 ml of distilled water. After that, K_2_HPO_4_·3H_2_O (1.0 g), KH_2_PO_4_ (1.0 g), NaHCO_3_ (10.0 g), NaCl (2.0 g), and resazurin solution (10 ml, 2.0 g resazurin dissolved in 10 ml double-distilled water) were added and stirred until dissolving, and then boiled distilled water was added to 1000 ml, mixed well, and cooled to room temperature. Then, tryptone (10.0 g), yeast extract (10.0 g), L-cysteine (1.0 g), and heme chloride solution (10 ml, 0.05 g heme chloride dissolved in 100 ml of 0.01 N NaOH solution) were supplemented. The pH was adjusted to 7.3 with NaOH test solution. All solution was autoclaved at 121 °C for 20 min and stored at 4 °C after cooling.

#### Preparation of Intestinal Flora Culture Solution

Six male Sprague–Dawley (SD) rats were provided by Shanghai Laboratory Animal Center (License No. SYXK (HU) 2013-0056, Shanghai China). Fresh intestinal contents (5.0 g) taken from SD rats were placed in a sterilized penicillin vial and mixed with normal saline at a ratio of 1 g:4 ml to make a suspension, and then the filtrate and anaerobic culture solution were mixed in a ratio of 1:9 to obtain enteric bacteria culture solution.

#### Sample Preparation

AR (1 mg/ml, 200 μL) was added to intestinal flora culture medium (1 ml), which was then filled with nitrogen without oxygen. The reactions were terminated by adding 1 ml n-butanol and 1 ml ethyl acetate after incubation for 0, 4, 8, 12, 24, 48, and 72 h, respectively. Next, the mixtures were vortexed for 5 min after adding 10 μL internal standard solution (nitrendipine, 2 μg/ml) and centrifuged at 14,000 rpm for 5 min. Subsequently, the organic phases were collected and evaporated under nitrogen gas, and 200 μL of methanol was added, vortexed, and centrifuged again at 14,000 rpm for 5 min, respectively. The supernatant was passed through the 0.22 μm millipore filter before injecting into the UPLC-Q-TOF-MS.

### Cell Membrane Immobilized Chromatography

A549 cells were purchased from Nanjing Kaiji biology Co., Ltd (Nanjing, China). A549 cells were seeded in a plastic culture flask containing DMEM, and then placed in a humidified incubator containing 5% CO_2_ at 37 °C. The medium was replaced every day until A549 cells grew to 80–90% confluence. A549 cells were starved in serum-free medium for 3 h after washing with PBS. AR sample was incubated on intestinal bacteria (2 × 10^−4^ g/ml) for 0, 4, 8, 12, 24, 48, and 72 h, and then the incubated solution was separately added into the A549 cells for 90 min, and was washed repeatedly with PBS until without detected component. The dissociation solution (10.95 g/L Na_2_HPO_4_ and 12.91 g/L citric acid aqueous solution) was immediately added into the treated A549 cells, which were incubated at 37 °C and 5% CO_2_ for 30 min to inactivate the cell effect target. Finally, the dissociation solution was collected.

A549 cells were digested with pancreatin and suspended into DMEM/high glucose medium. The cell suspension was centrifuged at 3,000 rpm for 2 min at 4 °C; the cell density (1 × 10^7^ cells/mL) was adjusted by PBS and then dissociated at room temperature for 1 h with dissociation solution. The cells were quickly placed at −80 °C for 20 min, and thawed in a thermostatic water bath at 37 °C for 10 min. The freezing–thawing process was repeated for 4 times and centrifuged at 2000 rpm for 20 min. Then, the dissociation solution and intracellular dissociation solution were collected and evaporated under nitrogen gas, and 200 μL of methanol was added, vortexed for 2 min, and centrifuged at 11,000 rpm for 10 min. Finally, the supernatant was passed through the 0.22 μm millipore filter before injecting into the UPLC-Q-TOF-MS.

### UPLC-Q-TOF-MS Analysis

Chromatographic analysis was performed on a LC-20AD UPLC system (Shimadzu Corporation, Kyoto, Japan) equipped with hybrid quadrupole time-of-flight tandem mass spectrometry (Triple TOF™ 5,600, AB SCIEX, Foster City, CA, USA) coupled with electrospray ionization (ESI) source. Chromatographic separation was performed on a ACQUITY UPLC HSS T3 column (50 mm × 2.1 mm, 1.8 μm). The flow rate was 0.3 ml/min, and the mobile phase consisted of solvent A (0.1% formic acid in water, v/v) and solvent B (acetonitrile) with the optimized gradient elution: 0∼2 min, 2%∼8% B; 2∼8 min, 8%∼20% B; 8∼12 min, 20%∼35% B; 12∼18 min, 35%∼60% B; 18∼24 min, 60%∼70% B; 24∼28 min, 70%∼80% B; 28∼28 min, 80%∼2% B; and 28∼30 min, 2%∼2% B. The column temperature was set at 30 °C. In order to get better analysis results, the mass spectrometer was conducted in electrospray and multiple reaction monitoring scanning mode, in negative ion modes. The optimized parameters for mass spectrometer were as follows: capillary voltage, 0.5 kV; ion source temperature, 100 °C; cone gas flow rate, 50 L/h; desolvation temperature, 400 °C; and desolvation gas flow, 800 L/h. The information-dependent acquisition techniques and dynamic background subtraction were used to reduce the impact of matrix interference.

### Statistical Analysis

All data are presented as the mean ± standard deviation. Data from mice and NHBE cells were statistically evaluated using *t*-test for pair-wise comparison. *p* < 0.05 was considered to be a significant difference, *p* < 0.01 was considered to be extremely significant difference, and *p* > 0.05 was considered to be no significant difference. All statistical analyses were performed using SPSS software (version 22.0, IBM, Chicago, IL, USA).

## Results

### Network Pharmacology Analysis of AR in the Treatment of Lung Cancer

#### Potential Targets and Components Prediction

**FIGURE 1:**
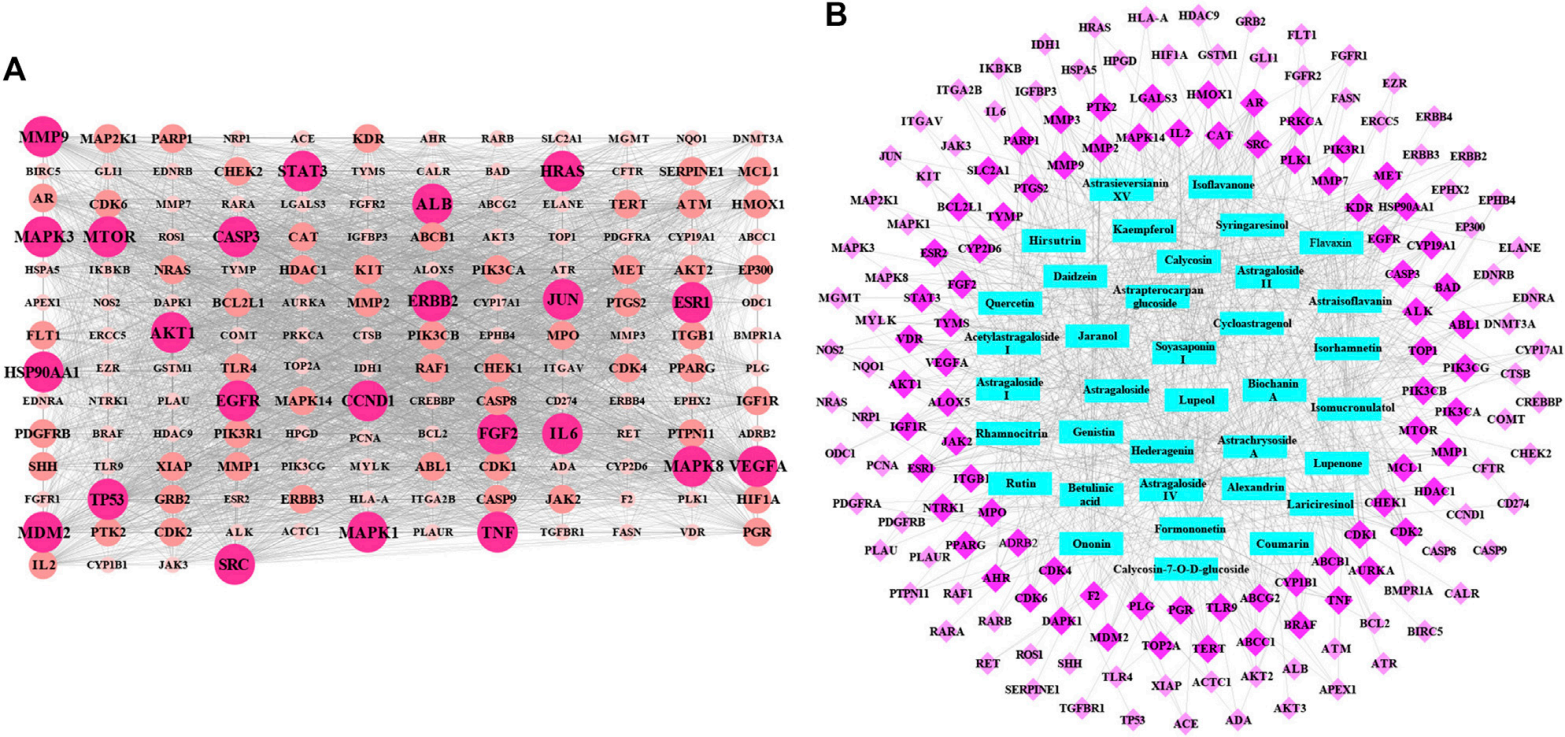
The protein–protein interaction (PPI) network and potential target-effective component network of AR in the treatment of lung cancer. **(A)** The PPI network of 160 potential targets. The node represents the potential target: the larger and brighter the node, the larger the interactions. **(B)** Network of 160 potential targets related to 36 potential effective components. The rose red diamond nodes represent 160 potential targets, blue rectangle nodes represent 36 potential effective components, and the brighter color diamond nodes represent the more important potential targets.

In this study, the computer virtual screening technology was used to provide a fast and efficient approach to obtain potential targets. At length, 160 potential targets of AR in the treatment of lung cancer were obtained, and the information of potential targets (degree ≥5) are listed in Table 1. As shown in Figure 1A, the PPI network consisted of 160 nodes and 3,720 edges (average node degree of 46.5 and average local clustering coefficient of 0.634), and the node represents the potential target; the “degree” value that indicated the strength of the potential target showed the larger the node, the brighter the color, and the larger the value. The results showed that the nodes of TP53, AKT1, VEGFA, EGFR, MAPK3, CCND1, HRAS, CASP3, SRC, ALB, JUN, STAT3, HSP90AA1, IL6, MAPK1, ESR1, ERBB2, TNF, MAPK8, MTOR, FGF2, and MMP9 are larger and brighter, indicating that they play a major role in the treatment of lung cancer. Further analysis found that these potential targets are mainly related to autophagy, apoptosis, and immune-mediated, cell cycle arrest and antioxidation. It is speculated that AR may play a role in the treatment of lung cancer through autophagy, apoptosis, and immune-mediated, cell cycle arrest and antioxidation.

**TABLE 1:** Information of potential targets of AR in the treatment of lung cancer (degree ≥ 5).

No.	Gene Name	Protein Name	Uniprot ID	Degree
1	ESR2	Estrogen receptor beta	Q92731	20
2	CDK1	Cyclin-dependent kinase 1	P06493	19
3	CDK2	Cyclin-dependent kinase 2	P24941	18
4	CYP19A1	Aromatase	P11511	18
5	CAT	Catalase	P04040	18
6	ESR1	Estrogen receptor	P03372	18
7	EGFR	Epidermal growth factor receptor	P00533	17
8	AR	Androgen receptor	P10275	16
9	IL2	Interleukin 2	P60568	16
10	ABCB1	ATP-dependent translocase ABCB1	P08183	15
11	HSP90AA1	Heat shock protein HSP 90-alpha	P07900	15
12	PARP1	Poly(ADP-Ribose) polymerase 1	P09874	15
13	TOP1	DNA topoisomerase I	P11387	14
14	KDR	Vascular endothelial growth factor receptor 2	P25968	14
15	SRC	Proto-oncogene tyrosine-protein kinase Src	P12931	14
16	ABCG2	ATP-binding cassette sub-family G member 2	Q9UNQ0	14
17	MAPK14	Mitogen-activated protein kinase 14	Q16539	13
18	MMP2	72 kDa type IV collagenase	P08253	13
19	MET	Hepatocyte growth factor receptor	P08581	12
20	ALOX5	Arachidonate 5-lipoxygenase	P09917	12
21	MMP3	Stromelysin-1	P08254	11
22	MMP9	Matrix metalloproteinase 9	P14780	11
23	TYMS	Thymidylate synthase	P04818	11
24	IGF1R	Insulin-like growth factor I receptor	P08069	11
25	TERT	Telomerase reverse transcriptase	O14746	11
26	CYP1B1	Cytochrome P450 1B1	Q16678	11
27	CHEK1	Serine/threonine-protein kinase Chk1	O14757	10
28	MCL1	Induced myeloid leukemia cell differentiation protein Mcl-1	Q07820	10
29	PIK3CG	Phosphatidylinositol 4,5-bisphosphate 3-kinase catalytic subunit gamma isoform	P48736	10
30	PRKCA	Protein kinase C alpha type	P17252	10
31	PTGS2	Prostaglandin G/H synthase 2	P35354	10
32	F2	Prothrombin	P00734	10
33	MMP1	Interstitial collagenase	P03956	9
34	ABL1	Tyrosine-protein kinase ABL1	P00519	9
35	ALK	ALK tyrosine kinase receptor	Q9UM73	9
36	FGF2	Fibroblast growth factor 2	P09038	9
37	STAT3	Signal transducer and activator of transcription 3	P40763	9
38	VDR	Vitamin D3 receptor	P11473	9
39	VEGFA	Vascular endothelial growth factor A	P15692	9
40	AKT1	RAC-alpha serine/threonine-protein kinase	P31749	9
41	MTOR	Serine/threonine-protein kinase mTOR	P42345	8
42	PIK3CA	Phosphatidylinositol 4,5-bisphosphate 3-kinase catalytic subunit alpha isoform	P42336	8
43	PLK1	Serine/threonine-protein kinase PLK1	P53350	8
44	HMOX1	Heme oxygenase 1	P09601	8
45	CYP2D6	Cytochrome P450 2D6	P10635	8
46	PPARG	Peroxisome proliferator activated receptor gamma	P37231	8
47	CDK6	Cyclin-dependent kinase 6	Q00534	8
48	ABCC1	Multidrug resistance-associated protein 1	P33527	8
49	AURKA	Aurora kinase A	O14965	7
50	LGALS3	Galectin-3	P17931	7
51	PLG	Plasminogen	P00747	7
52	MPO	Myeloperoxidase	P05164	7
53	PIK3CB	Phosphatidylinositol 4,5-bisphosphate 3-kinase catalytic subunit beta isoform	P42338	6
54	CASP3	Caspase-3	P42574	6
55	PIK3R1	Phosphatidylinositol 3-kinase regulatory subunit alpha	P27986	6
56	SLC2A1	Solute carrier family 2, facilitated glucose transporter member 1	P11166	6
57	BCL2L1	Bcl-2-like protein 1	Q07817	6
58	NTRK1	High affinity nerve growth factor receptor	P04629	6
59	ADRB2	Beta-2 adrenergic receptor	P07550	6
60	TLR9	Toll-like receptor 9	Q9NR96	6
61	TNF	Tumor necrosis factor	P01375	6
62	HDAC1	Histone deacetylase 1	Q13547	5
63	BAD	Bcl2-associated agonist of cell death	Q92934	5
64	MMP7	Matrix metalloproteinase 7	P09237	5
65	TYMP	Thymidine phosphorylase	P19971	5
66	JAK2	Tyrosine-protein kinase JAK2	O60674	5
67	ITGB1	Integrin beta-1	P05556	5
68	CDK4	Cyclin-dependent kinase 4	P11802	5
69	MDM2	E3 ubiquitin-protein ligase Mdm2	Q00987	5
70	TOP2A	DNA topoisomerase 2 alpha	P11388	5
71	PGR	Progesterone receptor	P06401	5
72	BRAF	Serine/threonine-protein kinase B-raf	P15056	5
73	PTK2	Focal adhesion kinase 1	Q05397	5
74	AHR	Aryl hydrocarbon receptor	P35869	5
75	DAPK1	Death-associated protein kinase 1	P53355	5

Based on the acquisition of potential targets, 36 potential effective components were obtained by matching potential targets, and their information is listed in Table 2. Potential target-effective component network was constructed by Cytoscape 3.6.1 software. As shown in Figure 1B, these 160 potential targets were associated with 36 potential effective components, and the blue nodes and rose red nodes represent 160 potential targets and 36 potential effective components, respectively. Potential target-effective component network indicated that the same component could act on multiple targets, and each target is usually associated with multiple components. These results suggested that different components in AR could regulate these same or similar targets to exert effect. So, in the study of TCM, as a complex system with multiple components and multiple targets, synergistic or antagonistic interactions among the different components of TCM should be considered.

**TABLE 2:** The summary of potential effective components of AR in the treatment of lung cancer.

Name	Molecular formula	CAS	Degree	Protein targets from effective components
Jaranol	C_17_H_14_O_6_	3301-49-3	42	ABCB1, ABCC1, ABCG2, ACTC1, AHR, AKT1, ALK, ALOX5, APEX1, AR, CAT, CDK1, CDK2, CDK6, CYP19A1, P1B1, DAPK1, EGFR, ESR1, ESR2, F2, IGF1R, KDR, KIT, MCL1, MET, MMP2, MMP3, MMP9, MYLK, NOS2, ODC1, ARP1, PIK3CG, PIK3R1, PLG, PLK1, PTGS2, PTK2, SRC, TERT, TOP2A
Flavaxin	C_17_H_20_N_4_O_6_	83-88-5	38	ABCG2, ABL1, ALK, ATM, AURKA, BMPR1A, BRAF, CDK1, CDK2, CDK4, CHEK1, EGFR, EPHB4, F2, HSP90AA1, JAK2, JAK3, JUN, KDR, MAPK1, MAPK14, MAPK8, MMP2, MMP3, MMP9, MTOR, NQO1, NTRK1, PDGFRB, PIK3CA, PIK3CB, PIK3CG, PPARG, PRKCA, PTK2, SRC, TGFBR1, TLR4
Astragaloside IV	C_41_H_68_O_14_	84687-43-4	37	ADRB2, AKT1, AKT2, AR, CAT, CDK1, CDK4, CYP2D6, EGFR, ESR1, F2, FGF2, HMOX1, HSP90AA1, IGF1R, IKBKB, IL2, ITGA2B, ITGAV, ITGB1, LGALS3, MDM2, MET, MMP1, MTOR, NRP1, PARP1, PIK3CA, PIK3CG, PLG, PKCA, SRC, STAT3, TOP1, TYMS, VDR, VEGFA
Isorhamnetin	C_16_H_12_O_7_	480-19-3	37	ABCB1, ABCC1, ABCG2, AHR, AKT1, ALK, ALOX5, APEX1, CAT, CDK1, CDK2, CDK6, CYP19A1, CYP1B1, DAPK1, EGFR, ESR1, ESR2, F2, HMOX1, IGF1R, KDR, MET, MMP2, MMP3, MMP9, MPO, MYLK, PARP1, PIK3CG, PIK3R1, PLG, PLK1, PTK2, SRC, TERT, TOP2A
Rhamnocitrin	C_16_H_12_O_6_	569-92-6	37	ABCB1, ABCC1, ABCG2, ADA, AHR, ALK, ALOX5, APEX1, AR, CAT, CDK1, CDK2, CDK6, CFTR, CYP19A1, CYP1B1, DAPK1, EGFR, ESR1, ESR2, F2, GSTM1, IGF1R, KDR, MCL1, MET, MMP2, MMP3, MMP9, MPO, PIK3CG, PI3R1, PLG, PLK1, PTGS2, SRC, TERT
Kaempferol	C_15_H_10_O_6_	520-18-3	35	ABCB1, ABCC1, ABCG2, ADA, AHR, ALK, ALOX5, CAT, CDK1, CDK2, CDK6, CFTR, CYP19A1, CYP1B1, DAPK1, EGFR, ESR1, ESR2, F2, IGF1R, KDR, MET, MMP2, MMP3, MMP9, MPO, PARP1, PIK3R1, PLK1, PTGS2, PTK2, SRC, TERT, TOP1, VEGFA
Quercetin	C_15_H_10_O_7_	117-39-5	35	ABCB1, ABCC1, ABCG2, AHR, AKT1, ALK, ALOX5, APEX1, CALR, CDK1, CDK2, CDK6, CYP19A1, CYP1B1, DAPK1, EGFR, ESR2, F2, IGF1R, KDR, MET, MMP2, MMP3, MMP9, MPO, MYLK, PARP1, PIK3CG, PIK3R1, PLK1, PTK2, SRC, TERT, TOP1, TOP2A
Astrachrysoside A	C_47_H_78_O_18_	123914-38-5	33	AR, CAT, CDK1, CDK2, CTSB, CYP2D6, ELANE, ESR1, FGF2, FGF2, FGFR2, HMOX1, HSP90AA1, IKBKB, IL2, ITGA2B, ITGAV, ITGB1, LGALS3, MAPK14, MET, MMP2, MMP9, MTOR, NTRK1, PIK3CA, PIK3CB, PPARG, SLC2A1, STAT3, TYMS, VDR, VEGFA
Astrapterocarpan glucoside	C_23_H_26_O_10_	94367-42-7	32	ABL1, AR, CASP3, CASP8, CAT, CDK1, CDK2, CHEK1, CYP19A1, EGFR, ERBB2, HMOX1, HSPA5, IL2, LGALS3, MAP2K1, MAPK1, MAPK14, MCL1, MGMT, MMP1, MMP2, MMP3, MMP9, ODC1, PARP1, PDGFRB, PTGS2, PTPN11, SLC2A1, TOP1, TYMP
Isomucronulatol	C_45_H_72_O_16_	84676-88-0	30	ABL1, ALK, AURKA, BAD, CASP3, CCND1, CDK1, CDK2, CHEK1, CYP19A1, EGFR, EP300, EZR, FLT1, HDAC9, HSP90AA1, KDR, MAPK1, MET, MMP1, MMP7, MTOR, PIK3CB, PIK3CG, PIK3R1, PLK1, PRKCA, RAF1, RET, SRC
Calycosin	C_16_H_12_O_5_	20575-57-9	30	ABCB1, ABCC1, ABCG2, ALK, ALOX5, BAD, BCL2, CDK2, CDK6, CHEK1, CYP19A1, CYP1B1, EGFR, ESR1, ESR2, F2, IGF1R, IGFBP3, IL2, KDR, MCL1, MET, PARP1, PLAU, PLAUR, PLG, PLK1, TERT, TLR9, TNF
Cycloastragenol	C_30_H_50_O_5_	84605-18-5	29	AKT1, AKT2, AKT3, ALOX5, AR, ATR, AURKA, BRAF, CDK2, CHEK1, EGFR, EPHX2, ERBB2, ESR1, ESR2, FGFR1, IGF1R, IKBKB, JAK2, KDR, MAPK14, MAPK3, MAPK8, MDM2, MMP3, MTOR, PGR, PLK1, ROS1
Formononetin	C_16_H_12_O_4_	485-72-3	27	ABCB1, ABCG2, AR, BAD, BCL2, CAT, CHEK1, CYP19A1, CYP1B1, EGFR, ERBB2, ERBB3, ERBB4, ERCC5, ESR1, ESR2, EZR, IDH1, IL2, MCL1, MMP2, MMP9, PPARG, RAF1, SRC, TLR9, TOP1
Lariciresinol	C_20_H_24_O_6_	27003-73-2	27	ABL1, ADA, AKT2, ALOX5, AURKA, BRAF, CDK1, CDK2, CDK4, CFTR, CHEK1, EPHB4, HDAC1, HIF1A, JAK2, MAP2K1, MCL1, MMP7, MTOR, NTRK1, PIK3CA, PIK3CB, PIK3CG, SLC2A1, TLR4, TOP1, XIAP
Ononin	C_22_H_22_O_9_	486-62-4	27	ABL1, ACE, CASP3, CD274, CYP19A1, EGFR, HDAC1, HRAS, IL2, KIT, MAPK14, MGMT, MMP1, MMP2, MMP3, MMP7, MMP9, NRAS, NTRK1, PARP1, PDGFRA, PPARG, PRKCA, SRC, TNF, TOP1, TYMP
Biochanin A	C_16_H_12_O_5_	491-80-5	26	ABCB1, ABCC1, ABCG2, ADRB2, BAD, BCL2, BRAF, CCND1, CHEK1, CHEK2, COMT, CYP19A1, CYP1B1, EGFR, ESR1, ESR2, HSP90AA1, IGFBP3, IL2, MCL1, NTRK1, PLAU, PPARG, RAF1, TERT, TLR9
Alexandrin	C_35_H_60_O_6_	474-58-8	25	ABL1, AKT1, ALK, ALOX5, AURKA, BCL2L1, FASN, FGF2, FGFR1, FLT1, HDAC1, HSP90AA1, IGF1R, IL2, JAK2, KIT, MAPK14, MET, PDGFRA, PDGFRB, PTPN11, RET, STAT3, TYMS, VEGFA
Astraisoflavanin	C_23_H_28_O_10_	131749-60-5	25	ABL1, BCL2L1, CASP3, CAT, CD274, CDK1, CDK2, CHEK1, ESR1, ESR2, GSTM1, HMOX1, HRAS, HSP90AA1, IGFBP3, MAPK8, MMP1, MMP7, PARP1, PIK3CA, PTGS2, SLC2A1, TOP2A, TYMP, TYMS
Betulinic acid	C_30_H_48_O_3_	472-15-1	25	ACE, AR, CAT, CYP17A1, CYP19A1, EDNRA, ESR2, IKBKB, ITGB1, MDM2, MMP1, MMP2, MMP3, NOS2, PGR, PPARG, PTGS2, PTPN11, RARA, RARB, TERT, TLR9, TOP1, TOP2A, VDR
Daidzein	C_15_H_10_O_4_	486-66-8	25	ABCB1, ABCC1, ABCG2, AR, BAD, BRAF, CAT, CDK6, CFTR, CYP19A1, CYP1B1, EGFR, ESR1, ESR2, HSP90AA1, IGFBP3, IL2, MCL1, NTRK1, PARP1, PLAU, PPARG, PTGS2, RAF1, TLR9
Soyasaponin I	C_48_H_78_O_18_	51330-27-9	25	ADRB2, AR, BCL2L1, CASP3, CASP9, CAT, CYP2D6, EDNRA, ESR2, F2, GLI1, GRB2, HLA-A, HMOX1, IGF1R, IL2, ITGB1, JUN, KDR, MMP9, PARP1, SRC, STAT3, TYMS,VDR
Genistin	C_21_H_20_O_10_	529-59-9	24	ABCB1, ABCG2, ALB, CDK2, CYP19A1, EGFR, ESR1, ESR2, HRAS, HSP90AA1, IL2, MAPK14, MGMT, MMP1, MMP2, MMP7, NRP1, PRKCA, PTGS2, SLC2A1, SRC, TNF, TOP1, TYMP
Astragaloside II	C_43_H_70_O_15_	84676-89-1	23	ABCB1, ADRB2, BCL2L1, CDK1, CDK2, CDK4, CTSB, CYP2D6, EGFR, F2, FGF2, HSP90AA1, KDR, LGALS3, MAPK14, MET, PPARG, PRKCA, STAT3, TOP1, TYMS, VDR,VEGFA
Isoflavanone	C_15_H_12_O_2_	4737-27-3	23	ABCG2, ACE, AURKA, CASP3, CAT, CDK1, CDK2, CREBBP, CTSB, CYP19A1, DNMT3A, ELANE, EP300, ESR1, ESR2, HMOX1, MAPK14, MCL1, MPO, PGR, PIK3CA, PIK3CB, TLR9
Astragaloside Ⅲ	C_41_H_68_O_14_	84687-42-3	22	ADRB2, AKT1, AR, CAT, CDK1, CDK2, CDK4, CDK6, CYP2D6, ESR1, ESR2, FGF2, FGFR1, HSP90AA1, IL2, KDR, LGALS3, RARB, STAT3, TYMS, VDR, VEGFA
Astrasieversianin XV	C_46_H_76_O_17_	101843-83-8	20	ADRB2, AKT1, AKT2, CDK1, CYP2D6, FGF2, FGFR1, HDAC1, HLA-A, HSP90AA1, IL2, MAPK14, MTOR, PIK3CA, PIK3CG, SLC2A1, STAT3, TOP1, TYMS, VEGFA
Hederagenin	C_30_H_48_O_4_	465-99-6	20	ALOX5, AR, CYP17A1, CYP19A1, EDNRA, EDNRB, ESR1, ESR2, IL6, ITGB1, MAPK3, MDM2, MMP1, MMP2, MMP3, NOS2, PGR, PTPN11, TERT, TOP1
Syringaresinol	C_22_H_26_O_8_	21453-69-0	19	ABCB1, AURKA, CDK1, CDK2, CHEK1, ERBB2, HDAC1, HDAC9, HIF1A, MCL1, MMP1, MTOR, NOS2, PCNA, PIK3CA, PIK3CB, PIK3CG, SERPINE1, TOP1
Acetylastragaloside I	C_47_H_74_O_17_	84687-47-8	17	ABCB1, AR, BCL2L1, CAT, CDK1, CYP2D6, ESR2, FGF2, HMOX1, HSP90AA1, LGALS3, PARP1, PRKCA, STAT3, TYMS, VDR, VEGFA
Lupeol	C_30_H_50_O	545-47-1	16	ABL1, AR, BIRC5, CYP17A1, CYP19A1, ESR1, ESR2, IGF1R, JAK2, KDR, MAPK14, MDM2, MPO, PRKCA, SHH, VDR
Calycosin-7-O-β-D-glucoside	C_22_H_22_O_10_	20633-67-4	14	ABCB1, ABL1, HRAS, HSP90AA1, IL2, MAPK14, MET, MGMT, PRKCA, SRC, TNF, TOP1, TYMP, TYMS
Rutin	C_27_H_30_O_16_	153-18-4	14	ABCG2, ACTC1, ALOX5, AR, CAT, CYP1B1, ESR2, IL2, PARP1, PLG, PTGS2, TERT, TNF, TP53
Astragaloside I	C_43_H_68_O_16_	84680-75-1	12	ABCB1, AR, BCL2L1, CAT, CDK1, CYP2D6, ESR2, FGF2, HMOX1, HSP90AA1, LGALS3, PARP1, PRKCA, STAT3, TYMS, VDR, VEGFA
Hirsutrin	C_21_H_20_O_12_	482-35-9	12	ABCG2, ALOX5, CAT, CYP1B1, ESR1, IL2, PARP1, PLG, PTGS2, SRC, TERT, TNF
Lupenone	C_30_H_48_O	1617-70-5	12	AKT1, AR, CAT, CYP17A1, CYP19A1, EPHB4, HPGD, KDRMAPK14, MPO, PDGFRB, PGR
Coumarin	C_9_H_6_O_2_	91-64-5	2	PARP1, TYMS

**TABLE 3:** Potential target-pathway enrichment of AR in the treatment of lung cancer.

Potential targets-signal pathway	Genes/ Term%	Nr. Genes	Potential target-signal pathway	Genes/Term%	Nr. Genes
Pathways in cancer	16.04	85	Progesterone-mediated oocyte maturation	20.20	20
PI3K-Akt signaling pathway	15.25	54	Transcriptional misregulation in cancer	10.75	20
Proteoglycans in cancer	23.38	47	Endometrial cancer	34.48	20
Human papillomavirus infection	12.12	40	Bladder cancer	48.78	20
Hepatitis B	23.93	39	Sphingolipid signaling pathway	15.97	19
MicroRNAs in cancer	13.04	39	mTOR signaling pathway	12.42	19
Human cytomegalovirus infection	16.44	37	T cell receptor signaling pathway	18.81	19
Human T-cell leukemia virus 1 infection	16.89	37	Insulin signaling pathway	13.87	19
Prostate cancer	38.14	37	p53 signaling pathway	25.00	18
MAPK signaling pathway	12.20	36	Axon guidance	9.94	18
Kaposi sarcoma–associated herpesvirus infection	19.35	36	Toll-like receptor signaling pathway	17.31	18
Focal adhesion	17.59	35	Fc epsilon RI signaling pathway	26.47	18
Ras signaling pathway	14.22	33	Measles	13.64	18
Human immunodeficiency virus 1 infection	15.57	33	Cell cycle	13.71	17
Rap1 signaling pathway	14.56	30	Osteoclast differentiation	13.28	17
FoxO signaling pathway	22.73	30	Th17 cell differentiation	15.89	17
Apoptosis	22.06	30	Apelin signaling pathway	11.68	16
Cellular senescence	18.75	30	Natural killer cell mediated cytotoxicity	12.21	16
Hepatocellular carcinoma	17.86	30	Herpes simplex virus 1 infection	8.65	16
Breast cancer	19.73	29	Platelet activation	12.10	15
Gastric cancer	19.46	29	NOD-like receptor signaling pathway	8.43	15
Viral carcinogenesis	13.93	28	IL-17 signaling pathway	16.13	15
HIF-1 signaling pathway	27.00	27	B cell receptor signaling pathway	21.13	15
Thyroid hormone signaling pathway	23.28	27	Cholinergic synapse	13.39	15
Hepatitis C	17.42	27	GnRH signaling pathway	15.05	14
Epstein-Barr virus infection	13.43	27	Oxytocin signaling pathway	9.15	14
Non-small cell lung cancer	40.91	27	Insulin resistance	12.96	14
Central carbon metabolism in cancer	41.54	27	cGMP-PKG signaling pathway	7.83	13
ErbB signaling pathway	30.59	26	Longevity regulating pathway	14.61	13
Relaxin signaling pathway	20.00	26	Gap junction	14.77	13
AGE-RAGE signaling pathway in diabetic complications	26.00	26	Non-alcoholic fatty liver disease (NAFLD)	8.72	13
Colorectal cancer	30.23	26	AMPK signaling pathway	10.00	12
Pancreatic cancer	34.67	26	Longevity regulating pathway	19.35	12
Melanoma	36.11	26	Serotonergic synapse	10.43	12
Chronic myeloid leukemia	34.21	26	Adrenergic signaling in cardiomyocytes	7.59	11
Fluid shear stress and atherosclerosis	18.71	26	Adherens junction	15.28	11
JAK-STAT signaling pathway	15.43	25	Fc gamma R-mediated phagocytosis	12.09	11
Glioma	33.33	25	Leukocyte transendothelial migration	9.82	11
Neurotrophin signaling pathway	20.17	24	Melanogenesis	10.89	11
Regulation of actin cytoskeleton	11.21	24	Adipocytokine signaling pathway	15.94	11
Estrogen signaling pathway	17.39	24	Pertussis	14.47	11
Small cell lung cancer	25.81	24	Thyroid cancer	29.73	11
C-type lectin receptor signaling pathway	22.12	23	NF-kappa B signaling pathway	10.53	10
Chagas disease (American trypanosomiasis)	22.33	23	Long-term potentiation	14.93	10
Toxoplasmosis	20.35	23	Parathyroid hormone synthesis, secretion and action	9.43	10
Tuberculosis	12.85	23	Leishmaniasis	13.51	10
Influenza A	13.45	23	Amebiasis	10.42	10
Renal cell carcinoma	33.33	23	Th1 and Th2 cell differentiation	9.78	9
Chemokine signaling pathway	11.58	22	Long-term depression	15.00	9
Phospholipase D signaling pathway	14.86	22	Type II diabetes mellitus	19.57	9
Signaling pathways regulating pluripotency of stem cells	15.83	22	Amyotrophic lateral sclerosis (ALS)	17.65	9
Prolactin signaling pathway	31.43	22	Epithelial cell signaling in Helicobacter pylori infection	13.24	9
Acute myeloid leukemia	33.33	22	Mitophagy	12.31	8
Autophagy	16.41	21	Apoptosis	24.24	8
VEGF signaling pathway	35.59	21	Regulation of lipolysis in adipocytes	14.55	8
Choline metabolism in cancer	21.21	21	Shigellosis	12.31	8
cAMP signaling pathway	9.43	20	Aldosterone-regulated sodium reabsorption	16.22	6
TNF signaling pathway	18.18	20			

#### Enrichment Analysis and Mechanism Prediction

**FIGURE 2:**
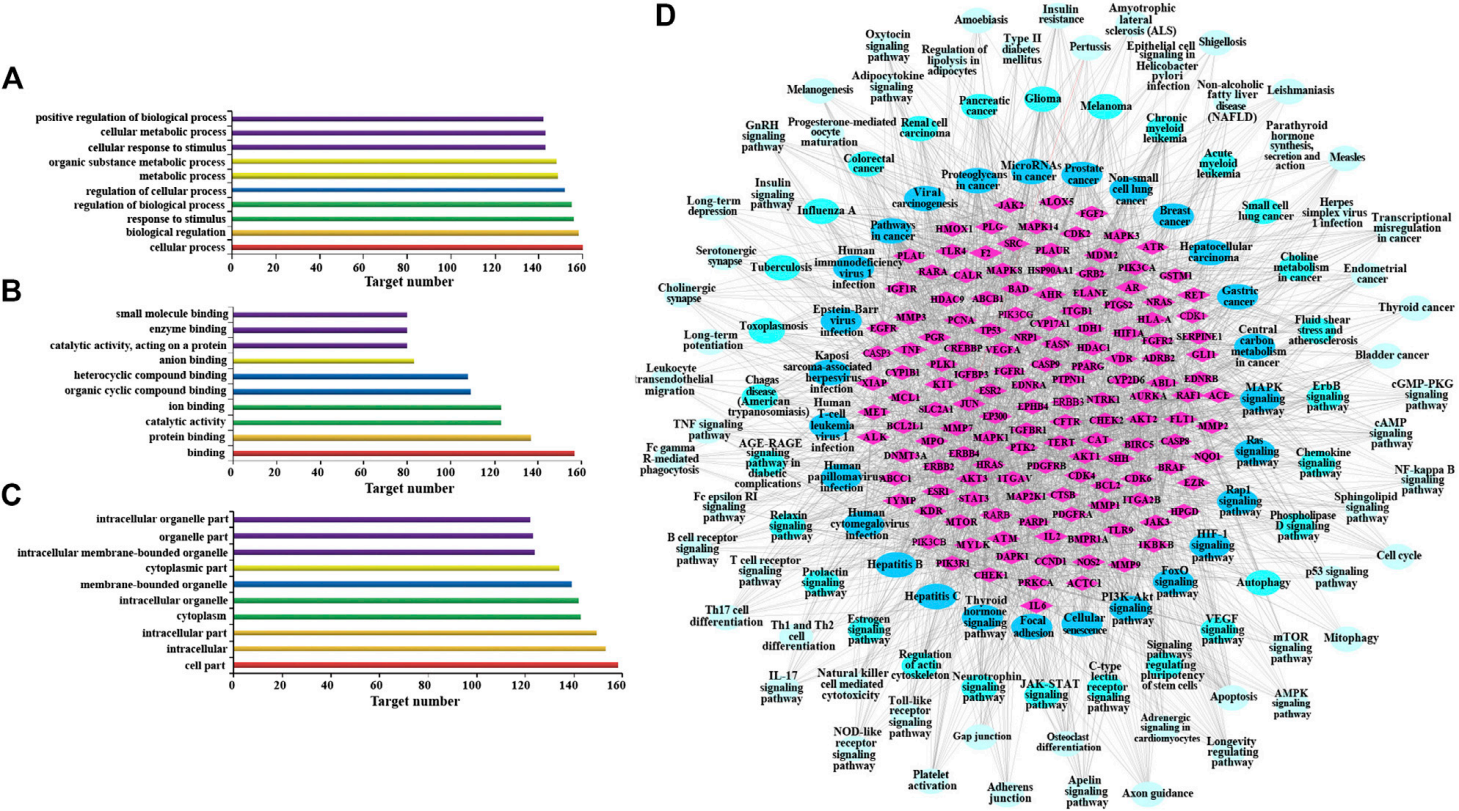
Gene Ontology (GO) enrichment analysis and Kyoto Encyclopedia of Genes and Genomes (KEGG) analysis of AR in the treatment of lung cancer. **(A)** Go enrichment analysis of potential targets in biological process. **(B)** Go enrichment analysis of potential targets in molecular function. **(C)** Go enrichment analysis of potential targets in cell composition. **(D)** The network of potential target-signal pathway by KEGG analysis (*p* ≤ 0.01). The diamond nodes represent potential targets, oval nodes represent signal pathways, and the brighter color oval nodes represent the more important the signal pathways.

Go enrichment analysis was performed on 160 potential targets, and limiting annotation was selected to homo sapiens. The top 10 terms of biological process (Figure 2A), molecular function (Figure 2B), and cell composition (Figure 2C) were selected. The results indicated that AR-regulated lung cancer mainly related to cellular process, biological regulation, response to stimulus, regulation of biological process, regulation of cellular process, and metabolic process, and mainly involved binding, protein binding, and catalytic activity in molecular function, and cell part, intracellular, intracellular part, cytoplasm, and intracellular organelle in cell composition.

KEGG analysis was performed using the ClueGO database in Cytoscape 3.6.1, and 115 KEGG pathways with *p*-value less than or equal to 0.01 were obtained (Table 3). In order to more intuitively show the relationship among potential targets and signal pathways, the potential target-signal pathway network was constructed (Figure 2D). According to the KEGG analysis, AR in the treatment of lung cancer was related to PI3K-Akt signaling pathway, MAPK signaling pathway, Ras signaling pathway, *etc*.

### Experimental Validation of AR in the Treatment of Lung Cancer *In Vivo*

#### Body Weight Change and Overall Appearance

**FIGURE 3:**
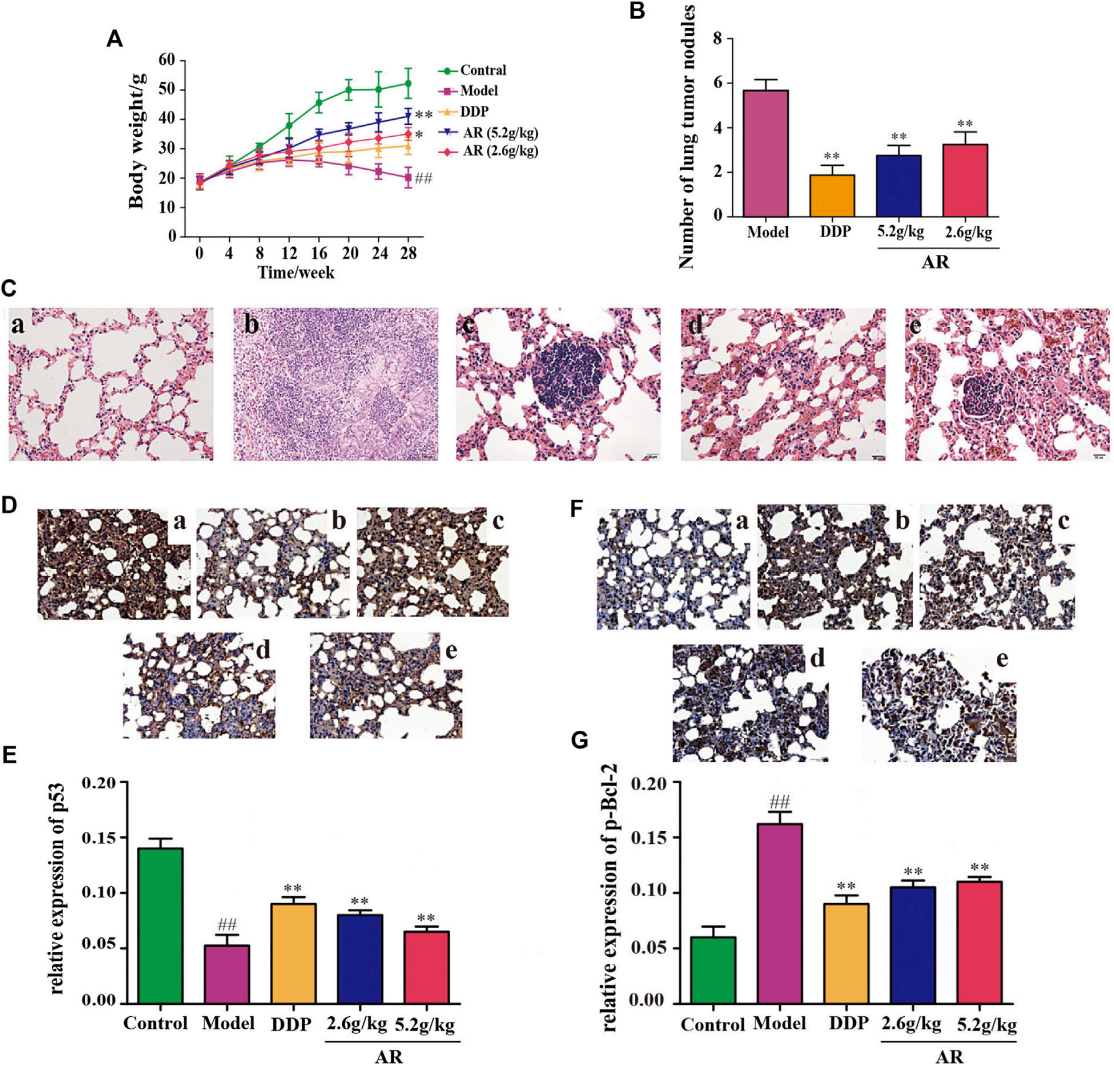
Experimental validation of AR in the treatment of lung cancer *in vivo*. **(A)** Body weight change of A/J mice in different groups. **(B)** The number of lung tumor nodules in different groups. **(C)** The HE staining of lung from A/J mice in different groups. a, control group; b, model group; c, DDP group; d, 5.2 g/kg AR group; e, 2.6 g/kg AR group. **(D)** and **(E)** IHC staining for p53 of lung from A/J mice in different groups. a, control group; b, model group; c, DDP group; d, 5.2 g/kg AR group. e, 2.6 g/kg AR group. **(F)** and **(G)** IHC staining for p-Bcl-2 of lung from A/J mice in different groups. a, control group; b, model group; c, DDP group; d, 5.2 g/kg AR group. e, 2.6 g/kg AR group. Compared with the model group, *p < 0.05, **p <0.01; compared with the control group, #*p* < 0.05, ##*p* < 0.01.

The body weight changes of the animals in different groups were shown in Figure 3A. Compared with the control group, the body weight of mice in other four groups were decreased, and mice in model group had the smallest body weight among these four groups (*p* < 0.01). Besides, compared with the model group, the body weight of 5.2 g/kg AR group and 2.6 g/kg AR group were significantly increased (*p* < 0.01, *p* < 0.05), and the most significant effect was observed in 5.2 g/kg AR group. The appearance of all mice were observations throughout the experimental period, and the results showed that the mice in model group suffered from nose bleeding and sparse neck hair, and no obvious symptoms in other four groups were observed.

#### Histopathological Study

After 28 weeks, lungs were removed from the mice for analysis. Except for the control group, what were observed in the other groups showed obvious lung lesions, tumor-like proliferation, and tumor nodules. Compared with the model group, the mice by DDP, 5.2 g/kg AR, and 2.6 g/kg AR treatment were able to significantly reduce (*p* < 0.01) the number of lung tumor nodules (Figure 3B). The HE staining and IHC staining were used to determine the success of the lung cancer model and the therapeutic effect of AR against lung cancer. As shown in Figure 3C, the HE staining results showed that lung tissues of mice in the control group had the intact structure, clear alveolar outline, thin alveolar septum, and no sign of inflammation, while the lung tissues disappear in the model group had serious damage to the alveolar structure, cancerous proliferation, and fibrosis, which indicates that the lung cancer model of A/J mice was successfully constructed. In addition, the administration of DDP and AR prevented the structural changes in the lung tissue and the infiltration of inflammatory cell, and improved lung tissue integrity. In addition, the above findings also suggest the effectiveness of AR in the treatment of lung cancer. The results of IHC staining were consistent with the HE staining results. In addition, the IHC staining results indicated that compared with the control group, the p53 expression (Figures 3D,E) was significantly decreased (*p* < 0.01), and the p-Bcl-2 expression (Figure 3F,G) was significantly increased (*p* < 0.01) in the lung tissue of model group. The administration of AR could upregulate the p53 expression and downregulate the p-Bcl-2 expression in the lung tissue, which indicated that AR can reverse the expression of p53 and p-Bcl-2 in lung cancer mice.

### Potential Targets and Mechanism Validation *In Vitro*

#### Astragali Radix Treatment Inhibited Cell Autophagy Induced by CSE

**FIGURE 4:**
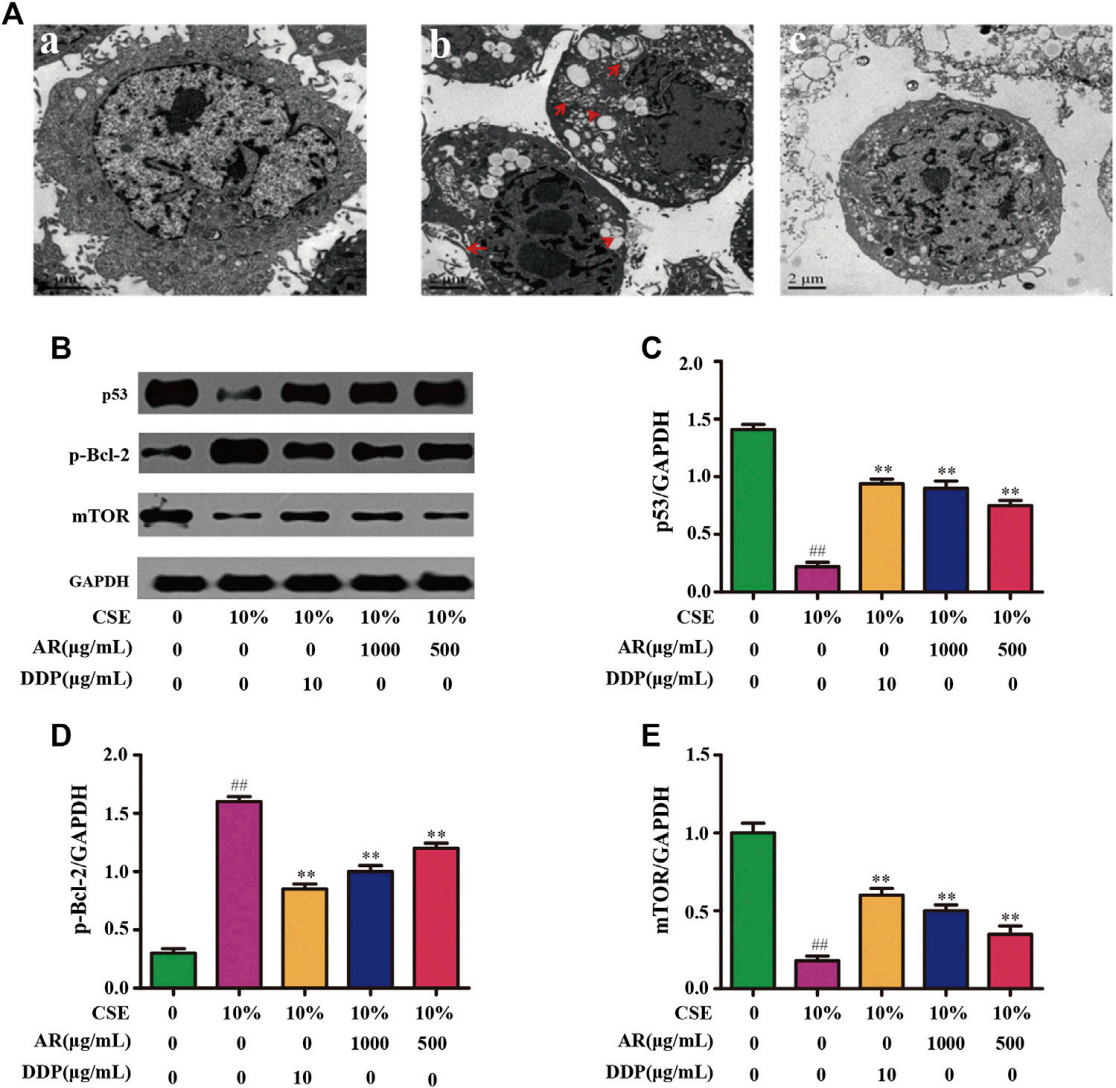
AR on cell autophagy by transmission electron microscope and p53, p-Bcl-2, mTOR expression in NHBE by Western blot analysis. **(A)** AR on cell autophagy by transmission electron microscope. a, control group; b, model group (autophagosomes or autolysosomes were indicated by the red arrows); c, 1000 µg/ml AR group. **(B)** The protein brands of p53, p-Bcl-2, mTOR and GAPDH. **(C)** p53 expression in NHBE by western blot analysis. **(D)** p-Bcl-2 expression in NHBE by western blot analysis. **(E)** mTOR expression in NHBE by western blot analysis. Compared with the model group, *p < 0.05, **p < 0.01; compared with the control group, #*p* < 0.05, ##*p* < 0.01.

CSE was used to induce autophagy in NHBE cells. The result showed that a large number of autophagy vacuoles appeared in the cytoplasm. Besides, autophagosome fuse with lysosome to form autolysosome, which decompose and destroy the organelles and damage the normal function of cells. After treatment with AR, the number of autophagosomes and autolysosomes in the cells was significantly reduced, and the structural integrity of the cells was increased, as shown in Figure 4A.

#### Astragali Radix on p53, p-Bcl-2 and Mammalian Target of Rapamycin Expression in NHBE Cells

**FIGURE 5:**
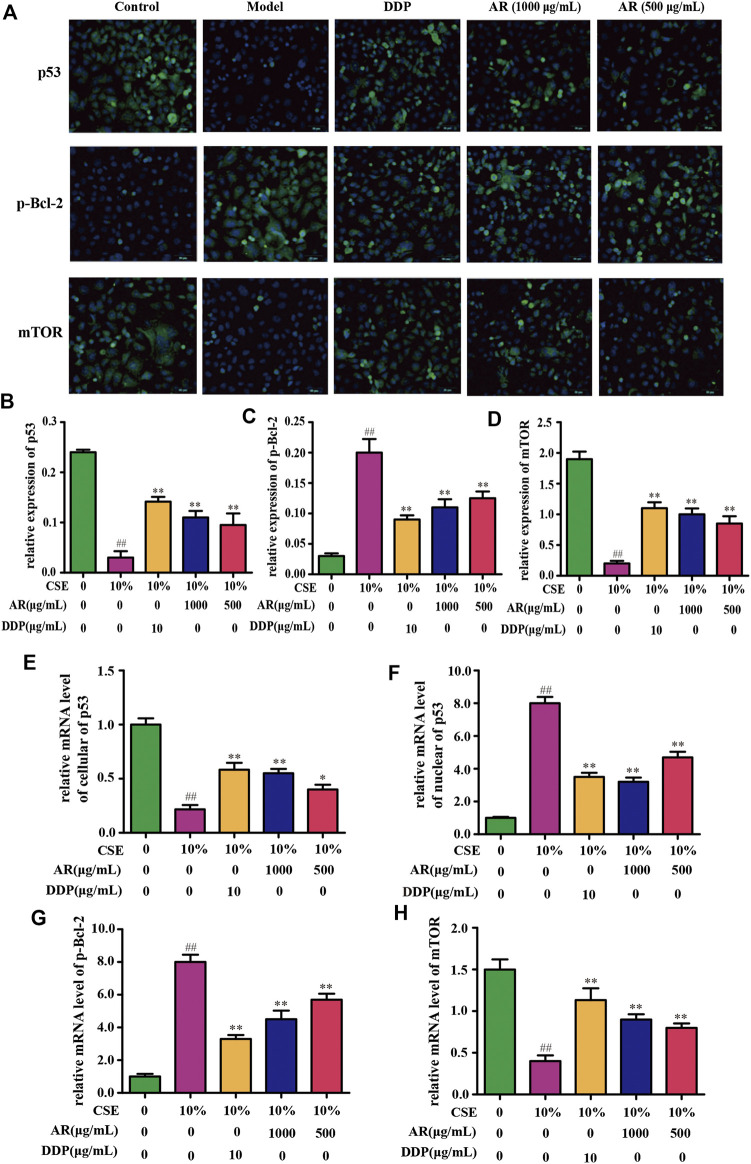
AR on p53, p-Bcl-2, and mTOR expression in NHBE cells by immunofluorescence staining and real-time PCR technology. **(A)** Representative pictures of p53, p-Bcl-2, and mTOR immunofluorescence in different groups. **(B) (C)** and **(D)** Relative p53, p-Bcl-2, and mTOR expression in NHBE by immunofluorescence staining. **(E)** and **(F)** Relative mRNA level of cellular and nuclear p53, respectively. **(G)** and **(H)** Relative mRNA level of p-Bcl-2 and mTOR, respectively. Compared with the model group, *p < 0.05, **p < 0.01; compared with the control group, #*p* < 0.05, ##*p* < 0.01.

The potential targets, p53, p-Bcl-2, and mTOR, predicted by network pharmacology technology, were verified at the protein and gene levels. As shown in Figures 4B,C, compared with the model group, the p53 expression in NHBE cells was significantly increased in the 1000 µg/ml AR group and 500 µg/ml AR group (*p* < 0.01). As shown in Figures 5A,B, immunofluorescence staining analyses showed that 1000 µg/ml AR and 500 µg/ml AR could increase the expression of p53 (*p* < 0.01). RT-PCR results (Figures 5E,F) showed that the relative mRNA level of cellular p53 has been increased (*p* < 0.01) and the relative mRNA level of nuclear p53 has been decreased (*p* < 0.01) by the treatment of 1000 µg/ml AR, respectively. The above results indicated that AR could promote p53 expression, thereby inhibiting autophagy and protecting cells from autophagy.

Western blot analysis (Figures 4B,D) and immunofluorescence (Figures 5A,C) showed that p-Bcl-2 expression was significantly reduced in NHBE cells after treatment with AR (*p* < 0.01). In addition, compared with model group, the relative mRNA level of p-Bcl-2 was significantly reduced (*p* < 0.01) in 1000 µg/ml AR group and 500 µg/ml AR group (Figure 5G). The above results indicated that AR could reduce the phosphorylation level of Bcl-2, inhibit autophagy, and protect cells.

As shown in Figures 4B,E, compared with the model group, mTOR expression in NHBE cells was significantly increased in the 1000 µg/ml AR group and 500 µg/ml AR group (*p* < 0.01). RT-PCR results (Figure 5H) showed that the relative mRNA level of mTOR has also been increased by the treatment of 1000 µg/ml AR and 500 µg/ml AR (*p* < 0.01). Immunofluorescence staining showed increased expression of mTOR expression in AR-treated cells at the concentration of 1000 µg/ml AR and 500 µg/ml AR (Figures 5A,D, *p* < 0.01). mTOR is a major negative regulator of autophagy and a key protein for controlling autophagy. The above results showed that AR could increase mTOR expression and regulate cell autophagy to protect cells.

#### Effect of Astragali Radix on p53/AMPK/Mammalian Target of Rapamycin Signaling Pathway

**FIGURE 6:**
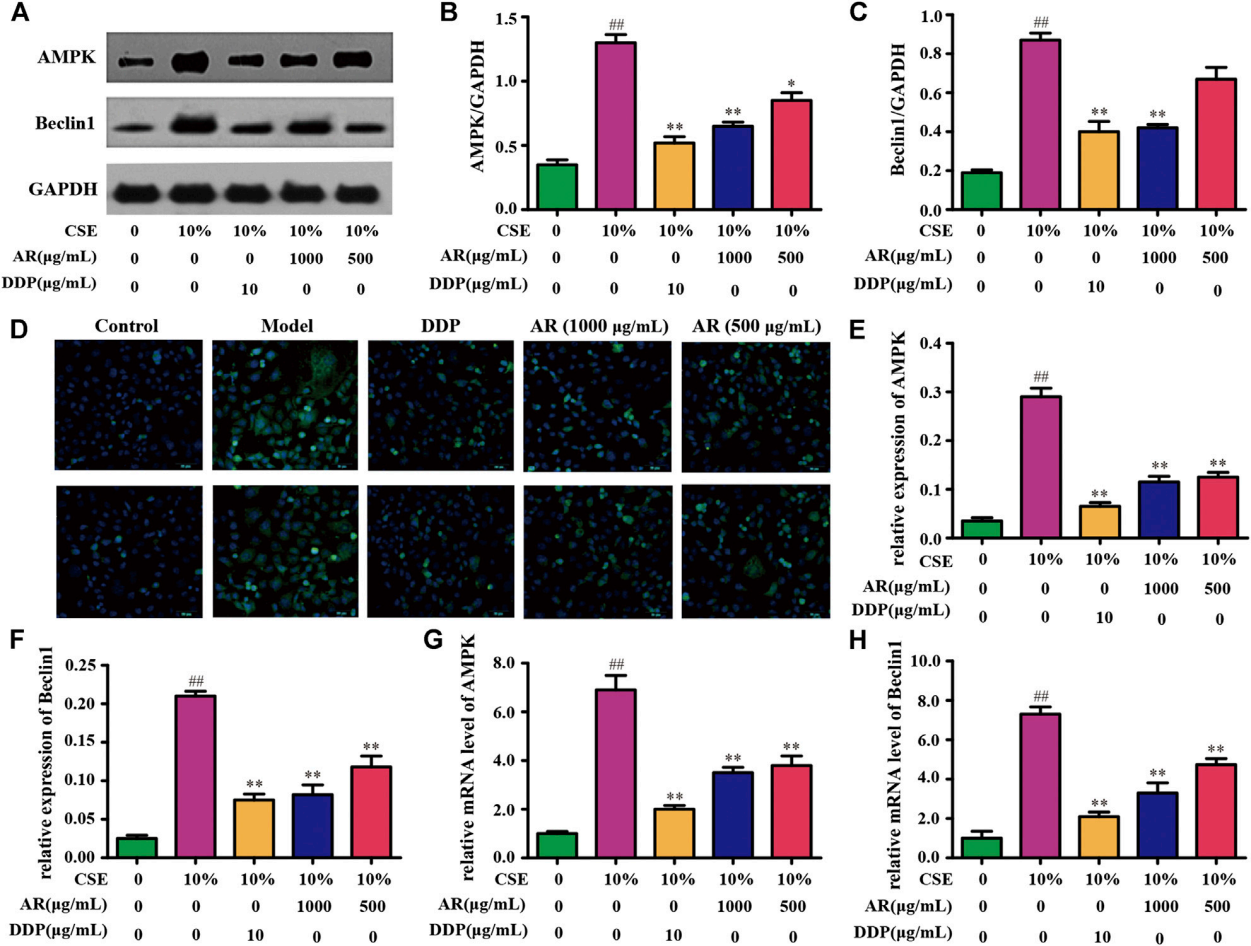
AR on AMPK and Beclin1 expression in NHBE cells. **(A)** The protein brands of AMPK, Beclin1, and GAPDH. **(B)** and **(C)** AMPK and Beclin1 expression in NHBE by Western blot analysis. **(D)** Representative pictures of AMPK and Beclin1 immunofluorescence in different groups. **(E)** and **(F)** Relative AMPK and Beclin1 expression in NHBE by immunofluorescence. **(G)** and **(H)** Relative mRNA level of AMPK and Beclin1, respectively. Compared with the model group, *p < 0.05, **p < 0.01; compared with the control group, #*p* <0.05, ##*p* < 0.01.

**FIGURE 7:**
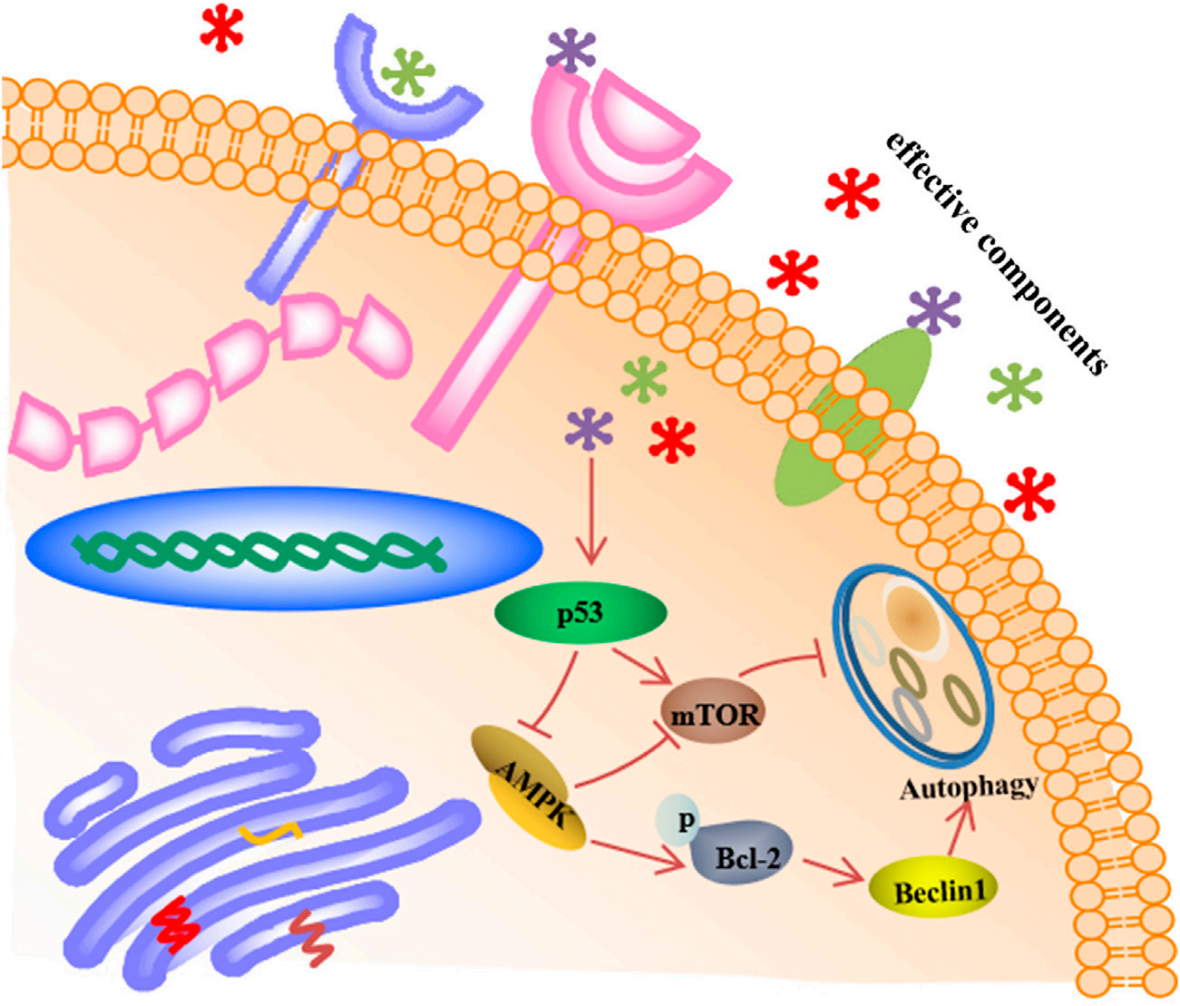
The p53/AMPK/mTOR signaling pathway of AR in the treatment of lung cancer by preliminary prediction.

According to the KEGG analysis, AR in the treatment of lung cancer was mainly related to PI3K-Akt signaling pathway. So, the expression of molecules downstream of the PI3K-Akt signaling pathway was mainly explored. Except for p53, p-Bcl-2, and mTOR (Figures 4,5), the expression of AMPK and Beclin1 at the protein and gene levels was also determined. As shown in Figure 6, compared with model group, Western blot analysis and immunofluorescence staining showed a significant reduction (*p* < 0.01) on AMPK and Beclin1 expression in NHBE cells after being treated with 1000 µg/ml AR. Besides, the relative mRNA level of AMPK and Beclin1 was significantly reduced (*p* < 0.01) in 1000 µg/ml AR group and 500 µg/ml AR group. It has been documented that p53 mediates autophagy through an AMPK/mTOR-dependent pathway (Tasdemir et al., 2008). AMPK activation leads to autophagy through negative regulation of mTOR and that many other factors involved in the autophagic process govern autophagy through AMPK/mTOR signaling (Jing et al., 2011). Based on the above research results, we speculate that AR in the treatment of lung cancer may through p53/AMPK/mTOR signaling pathway (Figure 7), but further validation is still required.

### Screening of Effective Components by Metabolism *in vitro* of Rat Intestinal Flora and Cell Membrane–Immobilized Chromatography

**FIGURE 8:**
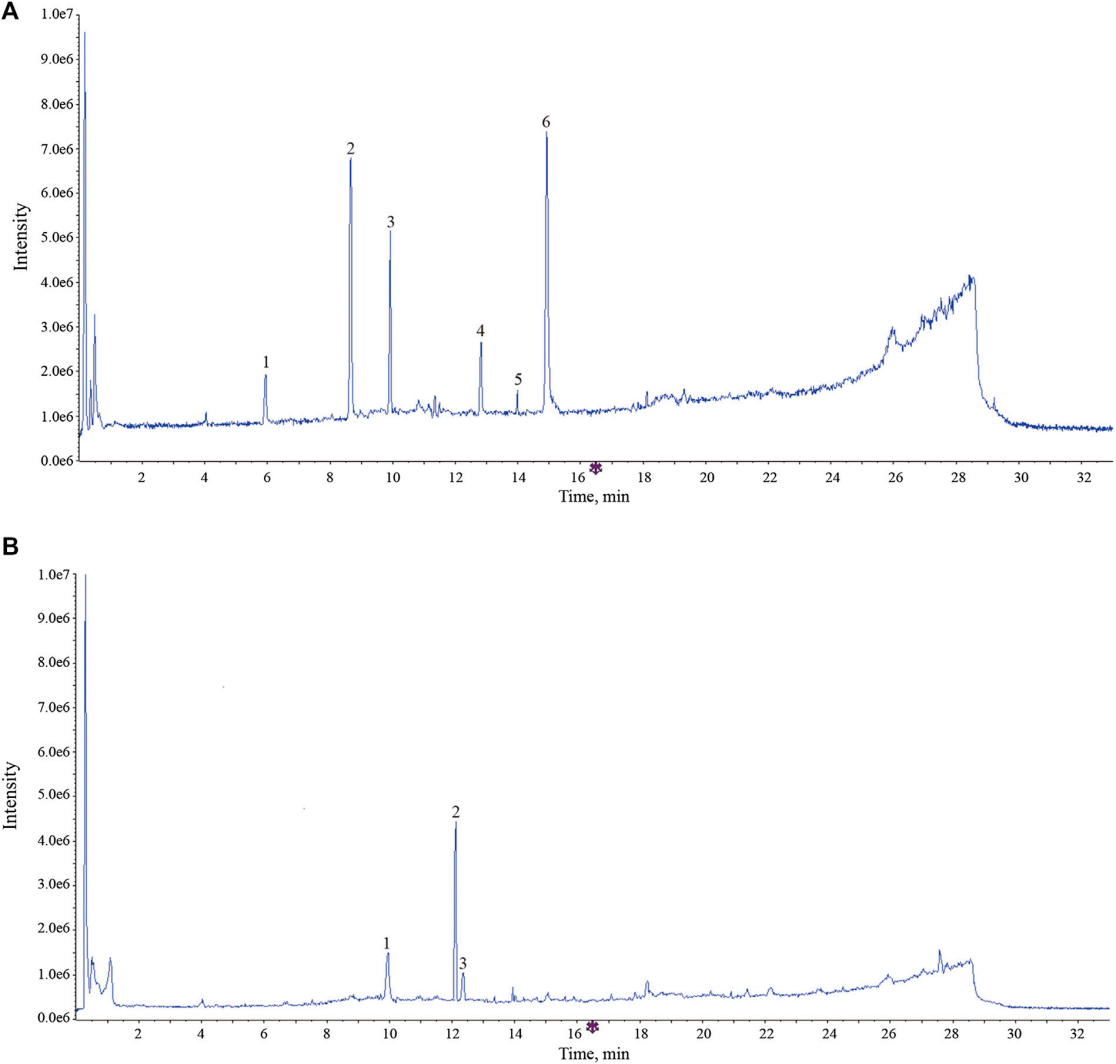
The typical total ion chromatogram of components bound to membrane proteins and intracellular protein. **(A)** Components bound to membrane proteins. 1, calycosin -7-O-β-D-glucoside; 2, ononin; 3, calycosin; 4, astragaloside IV; 5, metabolites of astragaloside II (M5); 6, cycloastragenol. **(B)** Components bound to intracellular protein. 1, calycosin; 2, formononetin; reduction product of calycosin (M9).

The UPLC-Q-TOF-MS analysis results indicated that these components in AR have undergone different degrees of metabolic transformation under the action of intestinal flora, and the typical total ion chromatogram of blank bacterial solution and the intestinal flora incubation solution of AR (2 h) as shown in Supplementary Figure S3. Detailed metabolite information was listed in Table 4, and the metabolic pathway mainly involves oxidation, reduction, hydrolytic deglycosylation, *etc*. The results of cell membrane–immobilized chromatography are shown in Figure 8. The six effective components, such as calycosin-7-O-β-D-glucoside, ononin, calycosin, astragaloside IV, metabolite of astragaloside II (M5), and cycloastragenol, can bind to cell membranes (Figure 8A). The three components, that is, reduction product of calycosin (M9), calycosin, and formononetin, can enter into the cell through the cell membrane by passive diffusion (Figure 8B).

**TABLE 4:** Identification of components in AR by using UPLC-ESI-Q-TOF-MS method in negative ion mode.

No.	RT (min)	Identified compounds	Element composition	Ionization	Prototype	Metabolic way
1	11.09	Astragaloside I	C_45_H_72_O_16_	[M+COOH]^-^	－	－
2	13.52	Astragaloside II	C_43_H_70_O_15_	[M+COOH]^-^	－	－
3	12.78	Astragaloside IV	C_41_H_68_O_14_	[M+COOH]^-^	－	－
4	9.83	Calycosin	C_16_H_12_O_5_	[M-H]^-^	－	－
5	6.04	Calycosin -7-O-β-D-glucoside	C_22_H_22_O_10_	[M+COOH]^-^	－	－
6	8.72	Ononin	C_22_H_22_O_9_	[M+COOH]^-^	－	－
7	13.64	M1	C_45_H_68_O_16_	[M-H]^-^	Astragaloside I	dehydrogenation
8	13.46	M2	C_36_H_60_O_10_	[M-H]^-^	Astragaloside I/Astragaloside II/Astragaloside IV	glycosylation
9	13.57	M3	C_39_H_62_O_11_	[M+COOH]^-^	Astragaloside I	glycosylation
10	14.97	M4	C_30_H_50_O_5_	[M+COOH]^-^	Astragaloside I/Astragaloside II/Astragaloside IV	glycosylation
11	13.89	M5	C_37_H_60_O_9_	[M+COOH]^-^	Astragaloside II	glycosylation+dehydroxylation
12	13.47	M6	C_35_H_58_O_9_	[M+COOH]^-^	Astragaloside IV	glycosylation
13	12.14	M7	C_16_H_12_O_4_	[M-H]^-^	Calycosin/Ononin	dehydroxylation
14	10.68	M8	C_16_H_16_O_5_	[M-H]^-^	Calycosin	hydrogenation+open loop
15	12.34	M9	C_16_H_16_O_4_	[M-H]^-^	Calycosin	hydrogenation+deoxidation
16	4.781	M10	C_22_H_22_O_11_	[M+COOH]^-^	Calycosin	hydrogenation+glucuronidation

Ultimately, these effective components, that is, calycosin-7-O-β-D-glucoside, ononin, calycosin, astragaloside IV, astragaloside II, cycloastragenol, and formononetin, together form the material basis of AR for prevention and treatment of lung cancer, and they come from the two-type components, flavone and saponin.

## Discussion

In this study, an integrated strategy for effective-component discovery of AR in the treatment of lung cancer was established. The results indicated that the integrated strategy can be applied to the efficiently screen effective components in complex systems. In our research on the bioactivity of AR, we found that the administered doses were high compared with single component drug. Therefore, the screening and confirmation of effective components in this article will help to reduce the dose by removing the ineffective components.

Modern pharmacological studies have shown that the activity of drugs is closely related to their cell membrane affinity and permeability. An important step in the role of TCM is the binding of effective components to cell membranes, specific enzymes, or receptors in cells. In this study, A549 cells were used as the separation carrier, AR was taken as the research object, and the specific affinity between each component in AR and cells was determined by cell membrane–immobilized chromatography. It is worth noting that the screening results show that calycosin-7-O-β-D-glucoside, ononin, calycosin, astragaloside IV, astragaloside II, cycloastragenol, and formononetin may be effective components of AR in the treatment of lung cancer, and which is consistent with the previous research results of AR in the prevention and treatment of cancer (He et al., 2013; Cheng et al., 2016; Xu et al., 2018). Through the ages, TCM have shown good efficacy in treating many and complex diseases (Sreenivasmurthy et al., 2017). The above research on the effective components of AR once again proved that the components of TCM are very complex and diverse, and multicomponent and multi-target may be the characteristic of TCM in treating diseases.

Autophagy has been implicated in a wide range of human diseases, including lung disorders such as lung cancer, chronic obstructive pulmonary disease, and lung infection diseases (Ryter and Choi, 2010; Ryter et al., 2012; Liu, et al., 2017; He et al., 2019). The article mainly investigated the mechanism of AR in the treatment of lung cancer from the perspective of autophagy according to the predictions of network pharmacology. The results suggested that AR may inhibit the development of lung cancer by reducing the p53 expression in the nucleus and promoting p53 expression in the cytoplasm, downregulating the level of p-Bcl-2 and promoting the autophagy inhibitory factor mTOR expression. In order to further explore the mechanism of AR in the treatment of lung cancer, except for the p53, p-Bcl-2 and mTOR, the expression of other molecules downstream of the PI3K-Akt signaling pathway was explored, including AMPK and Beclin1. Under stress, AMPK can promote the dissociation and phosphorylation of autophagy-related gene Bcl-2 (Zhou et al., 2011; He et al., 2012; Meng et al., 2015) and inhibit mTOR expression (Chen et al., 2015; Prietodomínguez et al., 2016; Yu et al., 2017), and ultimately promote the occurrence and development of autophagy. Beclin1 is an interacting protein of Bcl-2 (He et al., 2013); binding of Bcl-2 to Beclin1 inhibits Beclin1-mediated autophagy *via* sequestration of Beclin1 away from class III PI3K; and the interaction between Bcl-2 and Beclin1 is related to mTOR kinase-dependent phosphorylation of Bcl-2 (Levine et al., 2008; Pattingre et al., 2008; Chiang et al., 2018; Xu and Qin, 2019). The preliminary research results indicated that the regulation of autophagy may be a useful strategy in the treatment of lung cancer.

In this study, an integrated strategy for effective-component discovery of AR in the treatment of lung cancer was established, which provides a valuable reference mode for finding the effective components of TCM. In addition, preliminary research results indicated that AR in the treatment of lung cancer may through p53/AMPK/mTOR signaling pathway, which laid a foundation for further in-depth study of the mechanism of AR in lung cancer. Despite some promising results were obtained in the study, there are still several potential limitations to improve. First, we have to admit that network pharmacology virtual screening has some limitations, and the predicted effective components and potential targets may need to be further comprehensively verified through a variety of different technologies. Even though the study adopts an integrated research strategy combining network analysis and *in vitro*/vivo studies, it still could not avoid some false positives. Moreover, we found that the dosage of AR was very high compared with single-component drug. Therefore, it is necessary to further knock out invalid components of AR to reduce dosage. Finally, the preliminary research results indicated that AR in the treatment of lung cancer may be through p53/AMPK/mTOR signaling pathway; certainly, further experimental validation should be needed to confirm this hypothesis.

## Data Availability

The original contributions presented in the study are included in the article/Supplementary Material, and further inquiries can be directed to the corresponding authors.

## Abbreviations

AR, astragali radix; TCM, traditional Chinese medicine; DDP, cisplatin; GAPDH, glyceraldehyde-3-phosphate dehydrogenase; p53, protein 53; p-Bcl-2, phosphorylated B-cell lymphoma-2; mTOR, mammalian target of rapamycin; AMPK, AMP-activated protein kinase; PPI, protein–protein interactions; GO, gene Ontology; KEGG, kyoto encyclopedia of genes and genomes; CSE, cigarettes extract; HE, hematoxylin-eosin; IHC, immunohistochemistry; NHBE, primary normal human bronchial epithelial; UPLC-ESI-TO-MS, ultra-performance liquid chromatography–electrospray quadrupole time-of-flight mass spectrometry.

## References

[B1] Chen Y. C. Chen D. Liu S. J. Yuan T. Y. Guo J. Fang L. H. (2019). Systematic elucidation of the mechanism of genistein against pulmonary hypertension via network pharmacology approach. Int. J. Mol. Sci 20 (22), 5569 10.3390/ijms20225569 PMC6888439 31703458

[B2] Chen Z. T. Zhao W. Qu S. Li L. Lu X. Su F. (2015). PARP-1 promotes autophagy via the AMPK/mTOR pathway in CNE-2 human nasopharyngeal carcinoma cells following ionizing radiation, while inhibition of autophagy contributes to the radiation sensitization of CNE-2 cells. Mol. Med. Rep 12 (2), 1868–1876. 10.3892/mmr.2015.3604 25872765 PMC4463980

[B3] Cheng H. Ge X. Zhuo S. Gao Y. Zhu B. Zhang J. (2018). β-elemene synergizes with gefitinib to inhibit stem-like phenotypes and progression of lung cancer via down-regulating EZH2. Front. Pharmacol 9, 1413 10.3389/fphar.2018.01413 30555330 PMC6284059

[B4] Cheng X. D. Gu J. F. Yuan J. R. Feng L. Jia X. B. (2016). Suppression of A549 cell proliferation and metastasis by calycosin via inhibition of the PKC-α/ERK1/2 pathway: an *in vitro* investigation. Mol. Med. Rep 13 (6), 3709–3710. 10.3892/mmr.2016.4976 26956998 PMC4805104

[B5] Chiang W. C. Wei Y. Kuo Y. C. Wei S. Zhou A. Zou Z. (2018). High-throughput screens to identify autophagy inducers that function by disrupting Beclin 1/Bcl-2 binding. ACS Chem. Biol 13 (8), 2247–2260. 10.1021/acschembio.8b00421 29878747 PMC6250051

[B6] Fishilevich S. Zimmerman S. Kohn A. Iny Stein T. Olender T. Kolker E. (2016). Genic insights from integrated human proteomics in GeneCards. Database 2016, baw030 10.1093/database/baw030 27048349 PMC4820835

[B7] Gfeller D. Grosdidier A. Wirth M. Daina A. Michielin O. Zoete V. (2014). SwissTargetPrediction: a web server for target prediction of bioactive small molecules. Nucleic Acids Res 42, W32–W38. 10.1093/nar/gku293 24792161 PMC4086140

[B8] Gu S. Lai L. H. (2020). Associating 197 Chinese herbal medicine with drug targets and diseases using the similarity ensemble approach. Acta Pharmacol. Sin 41, 432–438. 10.1038/s41401-019-0306-9 31530902 PMC7470807

[B9] He C. Bassik M. C. Moresi V. Sun K. Wei Y. Zou Z. (2012). Exercise-induced BCL2-regulated autophagy is required for muscle glucose homeostasis. Nature 481 (7382), 511–515. 10.1038/nature10758 22258505 PMC3518436

[B10] He C. Zhu H. Li H. Zou M. Xie Z. (2013). Dissociation of Bcl-2-Beclin1 complex by activated AMPK enhances cardiac autophagy and protects against cardiomyocyte apoptosis in diabetes. Diabetes 62 (4), 1270–1281. 10.2337/db12-0533 23223177 PMC3609561

[B11] He H. Zhou X. Wang Q. Zhao Y. (2013). Does the couse of astragalus-containing Chinese herbal prescriptions and radiotherapy benefit to non-small-cell lung cancer treatment: a meta-analysis of randomized trials. Evid Based Complement Alternat Med, 2013, 426207 10.1155/2013/426207 24454494 PMC3878281

[B12] He Y. Liu H. Jiang L. Rui B. Mei J. Xiao H. (2019). miR-26 Induces apoptosis and inhibits autophagy in non-small cell lung cancer cells by suppressing TGF-β1-JNK signaling pathway. Front. Pharmacol 9, 1509 10.3389/fphar.2018.01509 30687089 PMC6333751

[B13] Jiang L. Wang W. He Q. Wu Y. Lu Z. Sun J. (2017). Oleic acid induces apoptosis and autophagy in the treatment of Tongue Squamous cell carcinomas. Sci. Rep 7 (1), 11277 10.1038/s41598-017-11842-5 28900281 PMC5595908

[B14] Jing K. Song K. S. Shin S. Kim N. Jeong S. Oh H. R. (2011). Docosahexaenoic acid induces autophagy through p53/AMPK/mTOR signaling and promotes apoptosis in human cancer cells harboring wild-type p53. Autophagy 7 (11), 1348–1358. 10.4161/auto.7.11.16658 21811093 PMC3242799

[B15] Ke B. Wu X. Yang Q. Huang Y. Wang F. Gong Y. (2019). Yi-qi-yang-yin-tian-sui-fang enhances cisplatin-induced tumor eradication and inhibits interleukin-7 reduction in non-small cell lung cancer. Biosci. Rep 39 (6), BSR20190052 10.1042/BSR20190052 31138762 PMC6597844

[B16] Levine B. Sinha S. Kroemer G. (2008). Bcl-2 family members: dual regulators of apoptosis and autophagy. Autophagy 4 (5), 600–606. 10.4161/auto.6260 PMC2749577 18497563

[B17] Li Y. H. Yu C. Y. Li X. X. Zhang P. Tang J. Yang Q. (2018). Therapeutic target database update 2018: enriched resource for facilitating bench-to-clinic research of targeted therapeutics. Nucleic Acids Res 46 (D1), D1121–D1127. 10.1093/nar/gkx1076 29140520 PMC5753365

[B18] Liu G. Pei F. Yang F. Li L. Amin A. D. Liu S. (2017). Role of autophagy and apoptosis in non-small-cell lung cancer. Int. J. Mol. Sci 18 (2), 367 10.3390/ijms18020367 PMC5343902 28208579

[B19] Lou J. S. Yan L. Bi C. W. Chan G. K. Wu Q. Liu Y. (2016). Yu Ping Feng San reverses cisplatin-induced multi-drug resistance in lung cancer cells via regulating drug transporters and p62/TRAF6 signalling. Sci. Rep 6 (1), 31926 10.1038/srep31926 27558312 PMC4997265

[B20] Meng F. Y. Ning H. Sun Z. X. Huang F. F. Li Y. C. Chu X. (2015). Ursolic acid protects hepatocytes against lipotoxicity through activating autophagy via an AMPK pathway. Journal of Functional Foods 17, 172–182. 10.1016/j.jff.2015.05.029

[B21] Pattingre S. Tassa A. Qu X. Garuti R. Liang X. H. Mizushima N. (2008). Bcl-2 antiapoptotic proteins inhibit Beclin 1-dependent autophagy. Cell 122 (6), 927–939 10.1016/j.cell.2005.07.002 16179260

[B22] Prietodominguez N. Ordonez R. Fernandez A. Garciapalomo A. Muntane J. Gonzalezgallego J. (2016). Modulation of autophagy by sorafenib: effects on treatment response. Front. Pharmacol 7, 151 10.3389/fphar.2016.00151 27375485 PMC4896953

[B23] Ru J. Li P. Wang J. Zhou W. Li B. Huang C. (2014). TCMSP: a database of systems pharmacology for drug discovery from herbal medicines. J. Cheminf 6 (1), 13 10.1186/1758-2946-6-13 PMC4001360 24735618

[B24] Ryter S. W. Choi A. M. (2010). Autophagy in the lung. Proc. Am. Thorac. Soc 7 (1), 13–21. 10.1513/pats.200909-101JS 20160144 PMC3137145

[B25] Ryter S. W. Nakahira K. Haspel J. A. Choi A. M. (2012). Autophagy in pulmonary diseases. Annu. Rev. Physiol 74 (4), 377–401. 10.1164/rccm.201512-2468SO 10.1164/rccm.201512-2468SO 22035347

[B26] Sreenivasmurthy S. G. Liu J. Y. Song J. X. Yang C. B. Malampati S. Wang Z. Y. (2017). Neurogenic traditional Chinese medicine as a promising strategy for the treatment of alzheimer's disease. Int. J. Mol. Sci 18 (2), 272 10.3390/ijms18020272 PMC5343808 28134846

[B27] Tasdemir E. Maiuri M. C. Galluzzi L. Vitale I. Djavaheri-Mergny M. D’Amelio M. (2008). Regulation of autophagy by cytoplasmic p53. Nat. Cell Biol 10, 676–687. 10.1038/ncb1730 18454141 PMC2676564

[B28] Torre L. A. Bray F. Siegel R. L. Ferlay J. Lortet-Tieulent J. Jemal A. (2015). Global cancer statistics, 2012. CA A Cancer J. Clin 65 (2), 87–108. 10.3322/caac.21262 25651787

[B29] Wang H. Zhang W. X. Cheng Y. T. Zhang X. Y. Xue N. N. Wu G. R. (2018). Design, synthesis and biological evaluation of ligustrazine-flavonoid derivatives as potential anti-tumor agents. Molecules 23 (9), 2187 10.3390/molecules23092187 PMC6225232 30200208

[B30] Wang S. Xu X. Hu Y. Lei T. Liu T. (2019). Sotetsuflavone induces autophagy in non-small cell lung cancer through blocking PI3K/Akt/mTOR signaling pathway *in vivo* and *in vitro* . Front. Pharmacol 10, 1460 10.3389/fphar.2019.01460 31920653 PMC6915081

[B31] Wang X. Shen Y. Wang S. Li S. Zhang W. Liu X. (2017). PharmMapper 2017 update: a web server for potential drug target identification with a comprehensive target pharmacophore database. Nucleic Acids Res 45, W356–W360. 10.1093/nar/gkx374 28472422 PMC5793840

[B32] Xiao Z. Wang C. Q. Zhou M. H. Hu S. S. Jiang Y. Huang X. R. (2019). Clinical efficacy and safety of Aidi injection plus paclitaxel-based chemotherapy for advanced non-small cell lung cancer: a meta-analysis of 31 randomized controlled trials following the PRISMA guidelines. J. Ethnopharmacol 228, 110–122. 10.1016/j.jep.2018.09.024 30243827

[B33] Xu F. Cui W. Q. Wei Y. Cui J. Qiu J. Hu L. L. (2018). Astragaloside IV inhibits lung cancer progression and metastasis by modulating macrophage polarization through AMPK signaling. J. Exp. Clin. Canc. Res 37 (1), 207 10.1186/s13046-018-0878-0 PMC6116548 30157903

[B34] Xu H. D. Qin Z. H. (2019). Beclin 1, Bcl-2 and autophagy. Adv. Exp. Med. Biol 1206, 109–126. 10.1007/978-981-15-0602-4_5 31776982

[B35] Xu H. Y. Zhang Y. Q. Liu Z. M. Chen T. Lv C. Y. Tang S. H. (2019). ETCM: an encyclopaedia of traditional Chinese medicine. Nucleic Acids Res 47 (D1), D976–D982. 10.1093/nar/gky987 30365030 PMC6323948

[B36] Xue R. Fang Z. Zhang M. Yi Z. Wen C. Shi T. (2012). TCMID: traditional Chinese medicine integrative database for herb molecular mechanism analysis. Nucleic Acids Res 41, D1089–D1095. 10.1093/nar/gks1100 23203875 PMC3531123

[B37] Yu Y. Hou L. Song H. Xu P. Sun Y. Wu K. (2017). Akt/AMPK/mTOR pathway was involved in the autophagy induced by vitamin E succinate in human gastric cancer SGC-7901 cells. Mol. Cell. Biochem 424 (1), 173–183. 10.1007/s11010-016-2853-4 27796683

[B38] Zhang H. W. Hu J. J. Fu R. Q. Liu X. Zhang Y. H. Li J. (2018). Flavonoids inhibit cell proliferation and induce apoptosis and autophagy through downregulation of PI3Kγ mediated PI3K/AKT/mTOR/p70S6K/ULK signaling pathway in human breast cancer cells. Sci. Rep 8 (1), 11255 10.1038/s41598-018-29308-7 30050147 PMC6062549

[B39] Zhou F. Yang Y. Xing D. (2011). Bcl-2 and Bcl-xL play important roles in the crosstalk between autophagy and apoptosis. FEBS J 278 (3), 403–413. 10.1111/j.1742-4658.2010.07965.x 21182587

[B40] Zhu X. Y. Guo D. W. Lao Q. C. Xu Y. Q. Meng Z. K. Xia B. (2019). Sensitization and synergistic anti-cancer effects of Furanodiene identified in zebrafish models. Sci. Rep 9 (1), 4541 10.1038/s41598-019-40866-2 30872660 PMC6418268

