# Supplementary material for: An Integrated Strategy for Effective-Component Discovery of Astragali Radix in the Treatment of Lung Cancer
Source: Front Pharmacol. 2021 Jan 14;11:580978. doi: 10.3389/fphar.2020.580978 (PMC7898675; doi:10.3389/fphar.2020.580978)
Supplement: Supplementary file 1 [file datasheet1.doc]

**Supplement 1**

Identification of components in AR by using UPLC-ESI-Q-TOF-MS method

| NO. | Identified compounds | Molecular formula | M (Da) | RT/min |
| --- | --- | --- | --- | --- |
| 1 | Calycosin-7-O-β-D-glucoside | C_22_H_22_O_10_ | 446.12 | 6.043 |
| 2 | Ononin | C_22_H_22_O_9_ | 430.13 | 8.715 |
| 3 | Calycosin | C_16_H_12_O_5_ | 284.07 | 9.826 |
| 4 | Formononetin | C_16_H_12_O_4_ | 268.07 | 12.141 |
| 5 | Astragaloside IV | C_41_H_68_O_14_ | 784.46 | 12.785 |
| 6 | Astragaloside II | C_43_H_70_O_15_ | 826.47 | 13.515 |
| 7 | Astragaloside I | C_45_H_72_O_16_ | 868.48 | 14.857 |
| 8 | Cycloastragenol | C_30_H_50_O_5_ | 490.71 | 14.974 |


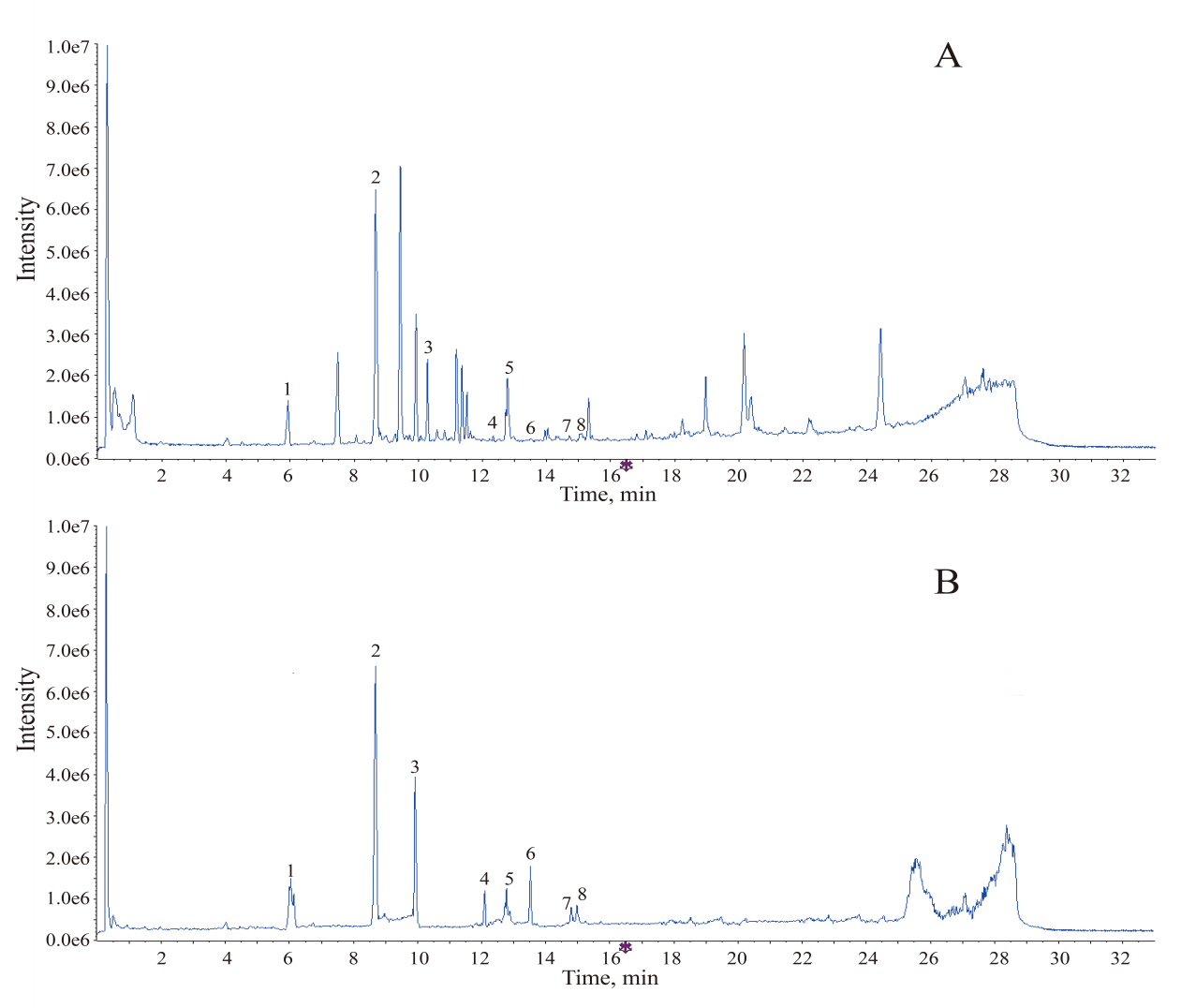


The typical total ion chromatograms of AR and reference substances in negative ion mode. (A) The typical total ion chromatograms of AR. (B) The typical total ion chromatograms of reference substances. 1, calycosin-7-O-β-D-glucoside; 2, ononin; 3, alycosin; 4, formononetin; 5, astragaloside IV; 6, astragaloside II; 7, astragaloside I; 8. cycloastragenol.
