# Supplementary figures and images for: An Integrated Strategy for Effective-Component Discovery of Astragali Radix in the Treatment of Lung Cancer
Source: Front Pharmacol. 2021 Jan 14;11:580978. doi: 10.3389/fphar.2020.580978 (PMC7898675; doi:10.3389/fphar.2020.580978)

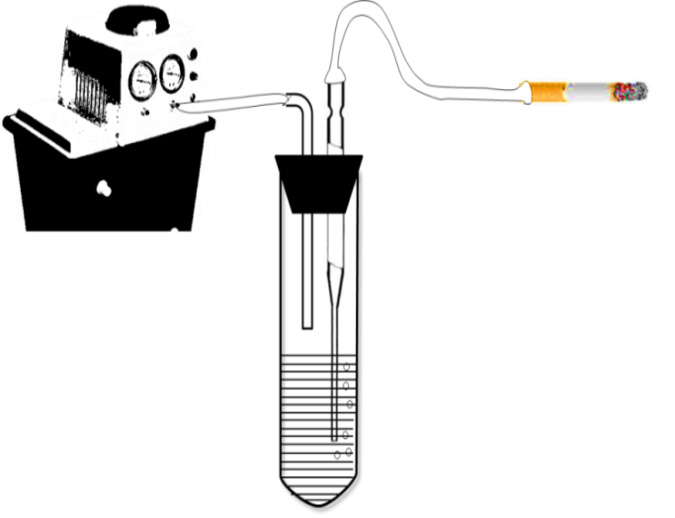

Supplement: Supplementary file 2 [file image1.jpeg]

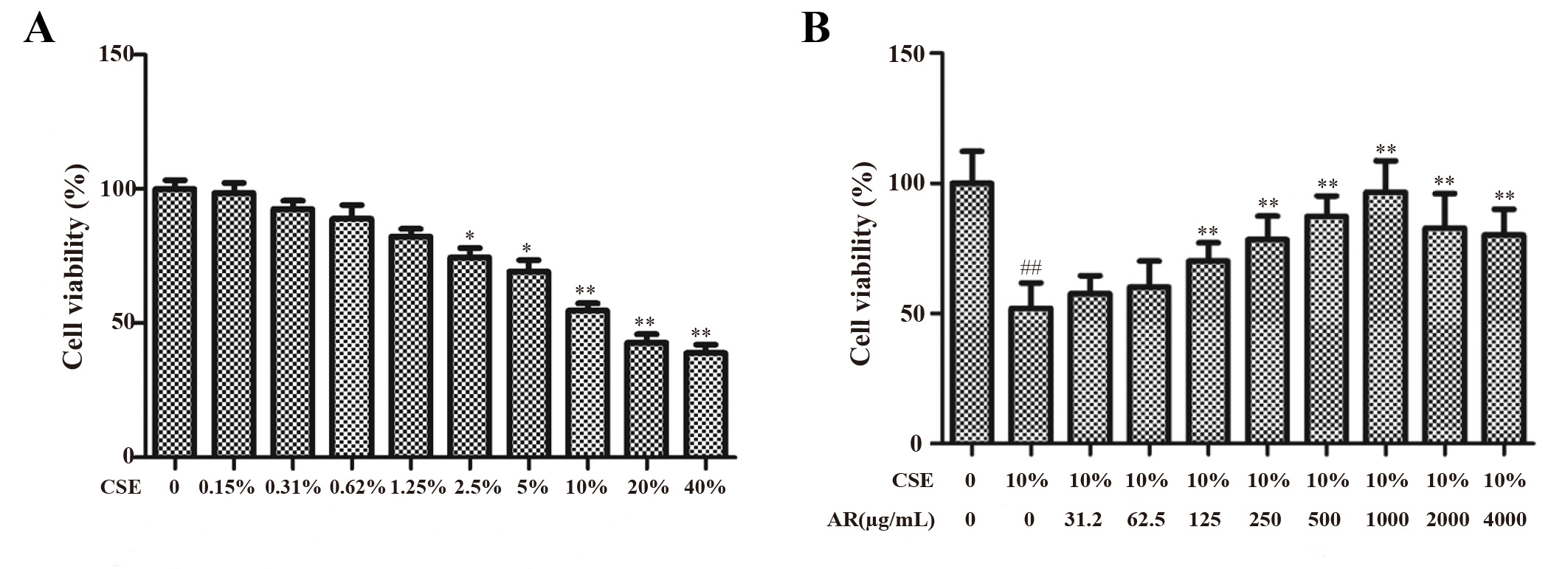

Supplement: Supplementary file 3 [file image2.jpeg]

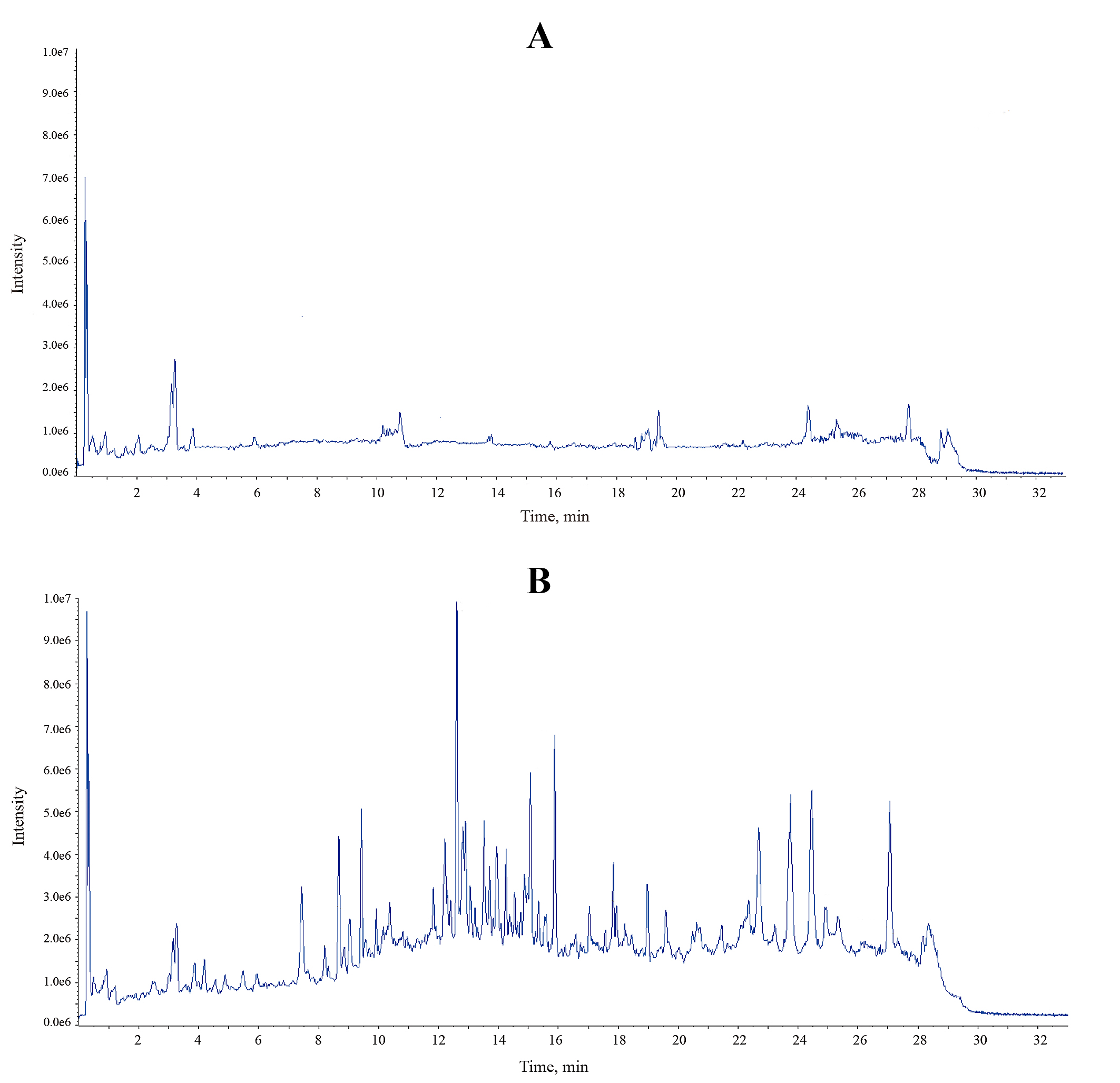

Supplement: Supplementary file 4 [file image3.jpeg]
